# Improved Trimethylangelicin Analogs for Cystic Fibrosis: Design, Synthesis and Preliminary Screening

**DOI:** 10.3390/ijms231911528

**Published:** 2022-09-29

**Authors:** Christian Vaccarin, Daniela Gabbia, Erica Franceschinis, Sara De Martin, Marco Roverso, Sara Bogialli, Gianni Sacchetti, Chiara Tupini, Ilaria Lampronti, Roberto Gambari, Giulio Cabrini, Maria Cristina Dechecchi, Anna Tamanini, Giovanni Marzaro, Adriana Chilin

**Affiliations:** 1 Department of Pharmaceutical and Pharmacological Sciences, University of Padova, Via Marzolo 5, 35131 Padova, Italy; 2 Center for Radiopharmaceutical Sciences ETH-PSI-USZ, Paul Scherrer Institute, 5232 Villigen, Switzerland; 3 Department of Chemical Sciences, University of Padova, Via Marzolo 1, 35131 Padova, Italy; 4 Department of Life Sciences and Biotechnology, University of Ferrara, Via Fossato di Mortara 74, 44121 Ferrara, Italy; 5 Center of Innovative Therapies for Cystic Fibrosis (InnThera4CF), University of Ferrara, Via Fossato di Mortara 74, 44121 Ferrara, Italy; 6 Department of Neurosciences, Biomedicine and Movement, Section of Clinical Biochemistry, University of Verona, Piazzale Stefani 1, 37126 Verona, Italy

**Keywords:** trimethylangelicin, synthesis, CFTR, pharmacokinetics, SEDDS

## Abstract

A small library of new angelicin derivatives was designed and synthesized with the aim of bypassing the side effects of trimethylangelicin (TMA), a promising agent for the treatment of cystic fibrosis. To prevent photoreactions with DNA, hindered substituents were inserted at the 4 and/or 6 positions. Unlike the parent TMA, none of the new derivatives exhibited significant cytotoxicity or mutagenic effects. Among the synthesized compounds, the 4-phenylderivative **12** and the 6-phenylderivative **25** exerted a promising F508del CFTR rescue ability. On these compounds, preliminary in vivo pharmacokinetic (PK) studies were carried out, evidencing a favorable PK profile *per se* or after incorporation into lipid formulations. Therefore, the selected compounds are good candidates for future extensive investigation to evaluate and develop novel CFTR correctors based on the angelicin structure.

## 1. Introduction

**Figure 1:**
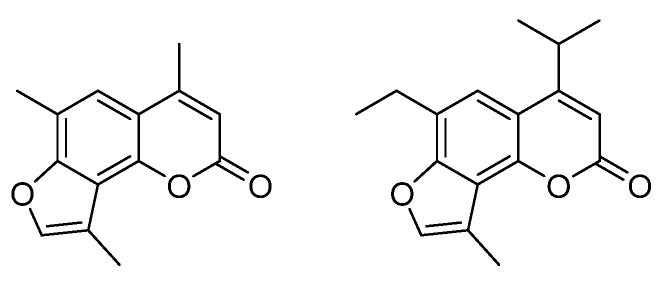
Structure of 4,6,4′-trimethylangelicin (TMA) and 4-isopropyl-6-ethyl-4′-methylangelicin (IPEMA).

Cystic fibrosis (CF) is an autosomal recessive disorder caused by mutations in the gene for the cystic fibrosis transmembrane conductance regulator (CFTR) protein, an anion-conducting transmembrane channel that regulates electrolyte transport and mucociliary clearance in the airways [1,2]. The recently approved drugs ivacaftor, lumacaftor, tezacaftor, and elexacaftor have changed the approach to CF therapy, improving the quality of life of CF patients [3]. These new drugs work on the CFTR defect, correcting or potentiating CFTR function [4,5,6]. TMA (4,6,4′-trimethylangelicin) (Figure 1) is a small molecule known to modulate the function of CFTR and control the inflammatory status of CF by targeting NF-κB and reducing IL-8 expression, demonstrating to be a promising agent for the treatment of CF [7,8,9]. Thus, TMA received an Orphan Drug Designation for the treatment of CF (EU/3/13/1137) by the EMA in 2013 [10].

Unluckily, TMA suffers from serious disadvantages, such as poor solubility, phototoxicity and mutagenicity. These drawbacks aroused a growing interest in investigating the structural modification of the TMA nucleus to identify new-generation TMA analogs with a comparable or better activity profile for the original hit compound but without the undesirable effects.

In previous work, we defined some structural determinants of the angelicin scaffold to maintain biological activities and eliminate side effects, thus identifying 4-isopropyl-6-ethyl-4′-methylangelicin (IPEMA) (Figure 1) as a promising candidate with TMA-like inhibitory activity on NF-kB/DNA interactions but lacking mutagenicity and DNA damaging properties [11]. Further investigations revealed that IPEMA significantly rescued F508del CFTR-dependent chloride efflux at nanomolar concentrations and maintained the potentiation activity of CFTR in FRT-YFP-G551D cells, acting by the same mechanism of TMA [12].

**Figure 2:**
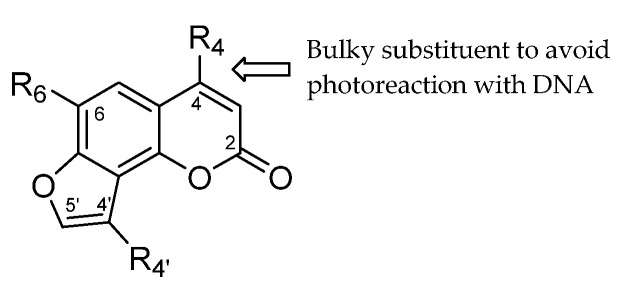
General structure of TMA analogs. See Table 1, Table 2 and Table 3 for the R specification.

Going on modifying the TMA scaffold, we report here the design, synthesis and preliminary screening of a small library of new TMA analogs (Figure 2). The pharmacokinetic profile of the most active derivatives, together with the previously reported IPEMA, was also investigated to identify the drug-likeness features to move TMA derivatives forward.

## 2. Results and Discussion

### 2.1. Chemistry

**Figure 3:**
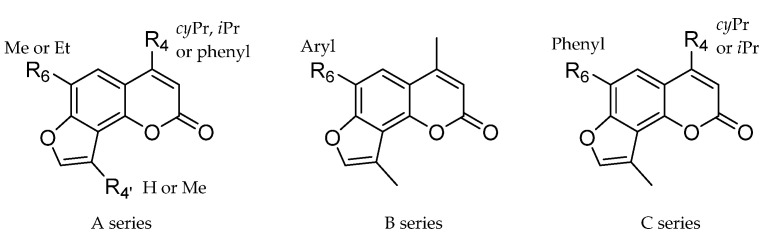
Series of TMA analogs.

As previously described, a hindered substituent at position 4 impairs DNA intercalation, thus preventing the unwanted photoreaction of the lactone or furan double bond of the furocoumarin scaffold with the nucleic acid [11,13]. For this reason, new analogs with cyclopropyl and aryl substituents at position 4 were designed (Figure 3, A series), in addition to the ethyl, propyl, and isopropyl derivatives already reported [11]. To also investigate the role of steric hindrance at position 6, we designed a further series of analogs with aryl substituents at this position (Figure 3, B series). Finally, derivatives with steric hindrances at both positions 4 and 6 were also considered (Figure 3, C series). In all cases, a 4′-methyl group or no substituent was inserted in the furan ring.

**Scheme 1:**
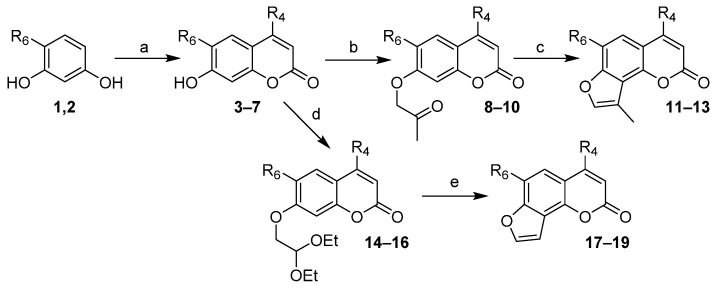
Synthesis of the A series of TMA analogs. Reaction conditions: a. Ethyl-3-cyclopropyl-3-oxopropionate (for **3**) or ethyl benzoylacetate (for **4**,**5**) or ethyl isobutyrylacetate (for **6**,**7**), H_2_SO_4_, 1 h, 40–95%; b. Chloroacetone, K_2_CO_3_, KI, acetone, reflux, 12 h, 40–95%; c. KOH, EtOH abs., reflux, 2 h, 10–33%. d. Chloroacetaldehyde diethylacetale, K_2_CO_3_, KI, DMF, reflux, 18 h, 36–83%; e. TFA, MW, 120 °C, 5 min, 24–29%. See Table 1 for the R specification.

Depending on the series of compounds, three different synthetic pathways were required. The A series, which included the analogs with steric hindrance only at position 4 was synthesized according to Scheme 1, adopting the previously reported synthetic strategies [11,14].

The starting 7-hydroxybenzopyran-2-ones (**3**–**7**) were synthesized by condensing the opportune resorcinol derivative (**1**, **2**) with the appropriate acetoacetic ester derivatives. Compounds **3**–**5** were functionalized with α-chloroacetone, obtaining the O-α-ketoethers **8**–**10**. The intermediate ethers were finally cyclized in an anhydrous alkaline medium that provided the desired 4′-methylangelicins **11**–**13**. Alternatively, starting benzopyranones **3**, **6**, and **7** were condensed with chloroacetaldehyde diethylacetale, affording ethers **14**–**16**, which were finally cyclized to angelicins **17**–**19** characterized by an unsubstituted furan ring.

**Scheme 2:**
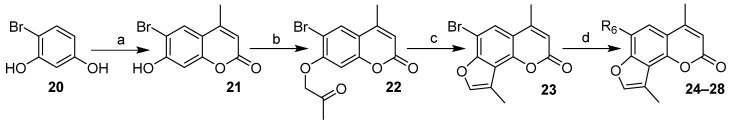
Synthesis of the B series of TMA analogs. Reaction conditions: a. Ethyl acetoacetate, H_2_SO_4_, 1 h, 82%; b. Chloroacetone, K_2_CO_3_, KI, acetone, reflux, 12 h, 69%; c. KOH, EtOH abs., reflux, 1 h, 34%; d. Arylboronic acid, Na_2_CO_3_, Pd(PPh_3_)_4_, DME, reflux, 2 h, 33–76%. See Table 2 for the R specification.

The B series, which included analogs with steric hindrance at position 6, was synthesized according to Scheme 2, using bromoangelicin **23** as a common intermediate.

The starting 6-bromo-7-hydroxybenzopyran-2-one (**21**) was synthesized by condensing bromoresorcinol (**20**) with ethyl acetoacetate, which was then functionalized with α-chloroacetone to O-α-ketoether **22** and finally cyclized to 4-methyl-6-bromo-4′-methylangelicins **23** in the same conditions as previously reported. The resulting 6-bromoangelicin was submitted to Suzuki coupling with opportune arylboronic acids to obtain the desired 6-arylangelicin (**24**-**28**). Benzene, pyridine and thiophene were chosen as representative aryl substituents; *p*- and *m*-aniline as more hydrophilic and salifiable groups were also introduced at position 6. Compound **25** was also prepared as a mesylate salt (**25a**, ***p*ANDMA mesylate** or ***p*ANDMAa**) to improve the poor solubility of the parent TMA and analogs.

**Scheme 3:**
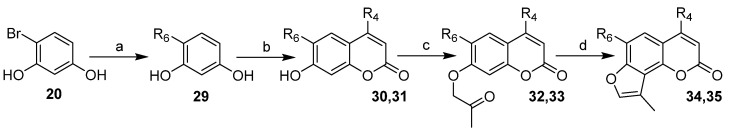
Synthesis of the C series of TMA analogs. Reaction conditions: a. Phenylboronic acid, Na_2_CO_3_, Pd(PPh_3_)_4_, DME, MW, 130 °C, 5 min, 26%; b. Ethyl isobutyrylacetate (for **30**) or ethyl-3-cyclopropyl-3-oxopropionate (for **31**), H_2_SO_4_, 1 h, 25%; c. Chloroacetone, K_2_CO_3_, KI, acetone, reflux, 6 h, 95%; d. KOH, EtOH abs., reflux, 1 h, 20–22%. See Table 3 for the R specification.

The C series, which included analogs with steric hindrance at both positions 4 and 6, was synthesized according to Scheme 3, using phenylresorcinol as a key intermediate.

In this case, the 6-phenyl substituent was inserted via Suzuki coupling on the starting resorcinol **20**, since we noted that Pechmann condensation on bromoresorcinol with hindered acetoacetic esters did not occur. The obtained 6-phenylresorcinol (**29**) was condensed with the opportune derivatives of ethyl acetoacetate, then functionalized with α-chloroacetone and finally cyclized to 4-alkyl-6-phenyl-4′-methylangelicins (**34**, **35**).

### 2.2. Biological Activity

#### 2.2.1. Functional Rescue of Mutant F508del CFTR Protein in CF Airway Cells

To investigate the corrective properties of the new TMA derivatives, we performed functional experiments to test whether some analogs maintain the F508del CFTR rescue property of the parent TMA.

To verify the possibility that the title compounds may act as correctors in CFBE41o-, overexpressing F508del and the high sensitivity halide-sensing yellow fluorescent protein (HS-YFP) YFP-H148Q/I152L, we examined the effect of 48 h preincubation with vehicle or the TMA analogs or TMA or VX809 (Lumacaftor), as a positive control.

**Figure 4:**
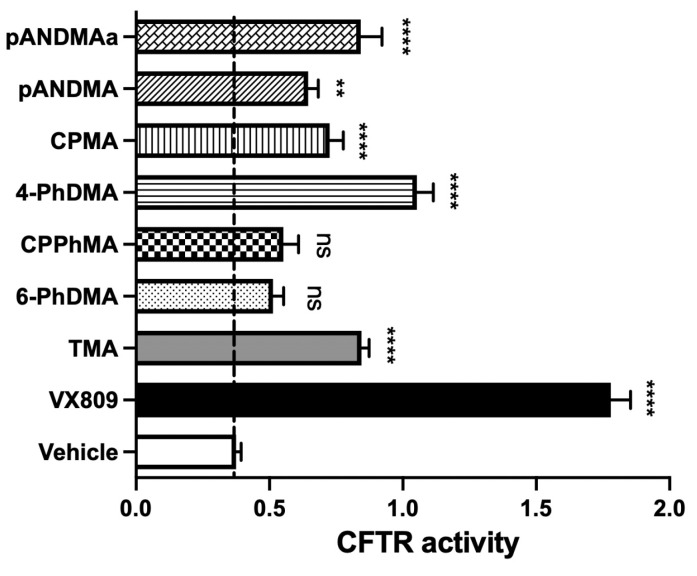
Evaluation of different TMA analogs as F508del CFTR correctors in CFBE41o- YFP-F508del cells. Correction of F508del-CFTR function was assessed, in a single cell, by measuring the decrease in YFP fluorescence upon addition of extracellular iodide and with the activating cocktail, in the presence or absence of CFTR inhibitor, CFTRInh-172. Iodide influx was tested under control condition (vehicle) or after 48 h incubation with the correctors 200 nM or 1 μM VX809. The YFP fluorescence decay rate was calculated by fitting the fluorescence data with an exponential function of time courses. CFTR activity was obtained by the difference between the fluorescence decay rate of YFP in the presence and absence of CFTRInh-172. Fluorescence coming from every single cell of at least five cells per field was recorded. Each bar corresponds to the mean ± SEM of data points coming from at least three different experiments (5–10 different cells/each experiment). Statistical comparisons were made using a nonparametric Kruskall–Wallis test (** *p* < 0.01, and **** *p* < 0.0001, ns: not significant). **pANDMAa** = **pANDMA mesylate**.

At the time of the assay, the correction of the F508del-CFTR function was evaluated in single-cell analysis by measuring the decrease in YFP fluorescence after the concomitant addition of extracellular iodide and the activating cocktail (20 μM forskolin and 5 μM VX770), in the presence or absence of the 10 μM CFTR inhibitor, CFTRinh-172. Figure 4 shows the activity of the TMA analogs compared to VX809 and TMA. Analogs not represented in the figure were less active than the vehicle. At least two derivatives show CFTR activity similar to that of the parent TMA.

Combining these data and analogous data obtained from the previous set of TMA derivatives [11,12], it can be seen that rescue activity was maintained, also hindering the 4 or 6 positions alternatively, taking TMA as a reference. The steric hindrances at positions 4 and 6 simultaneously have a detrimental effect depending on the dimension of the two substituents. If one of the substituents is a phenyl group, the other must be at most a methyl group, as can be seen by comparing compound **4-PhDMA** (active) with **4-PhEMA** (inactive, Figure 4). The presence of a 6-aryl substituent is poorly tolerated, as in the case of **6-PhDMA**, **mANDMA**, **PyDMA**, **ThiDMA**, **IPPhMA**, and **CPPhMA** (inactive), unless an amine is present specifically at the para position (compare **pANDMA** with **mANDMA**). A moderate steric hindrance at both positions 4 and 6 is allowed only if the substituents are small alkyl groups: this is the case of the previously identified **IPEMA** [12], bearing an isopropyl and an ethyl group, respectively. Almost all the derivatives without substituents in the furan ring exhibited activities lower than those of the vehicle.

**Figure 5:**
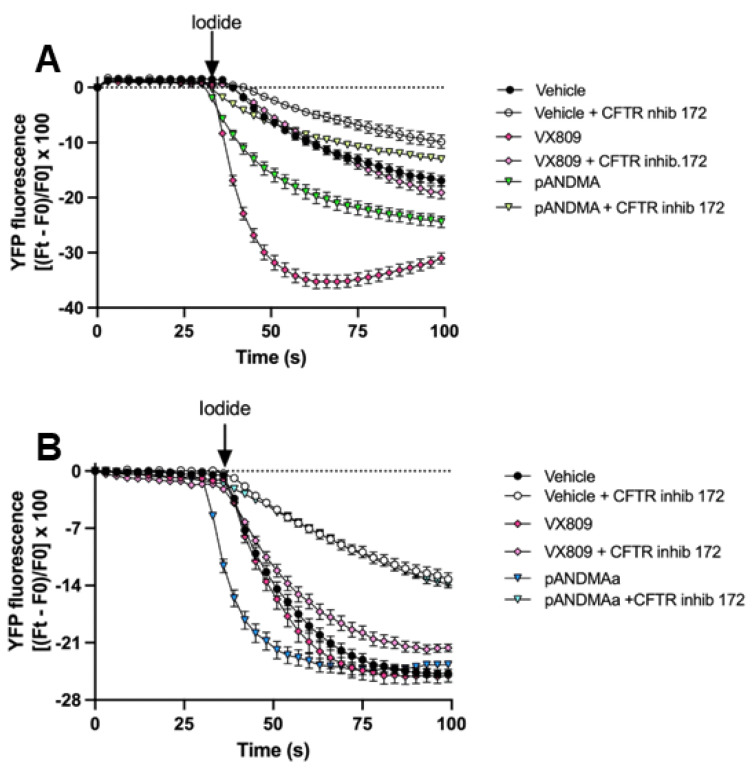
Representative time courses. Cells were treated for 48 h with vehicle or VX809 or **4-PhDMA** (**A**) or **pANDMAa** (**pANDMA mesylate**) (**B**). CFTR activity was measured as described in Figure 3. The arrow indicates the addition of iodide.

As shown in the representative experiments, correction of F508del-CFTR upon treatment with **4-PhDMA** and **pANDMA mesylate** (Figure 5, panels A and B, respectively) resulted in a greater reduction in YFP fluorescence induced by increased iodide influx compared to cells treated with vehicle alone.

Based on this preliminary screening, compounds **4-PhDMA** and **pANDMA mesylate** were the most interesting of the new series.

Differently from **IPEMA** [12], none of these TMA analogs were able to potentiate the CFTR function (data shown in Supplementary Materials, Figure S1).

#### 2.2.2. In Vitro Toxicity Studies: Photochemical Behavior

To confirm that the phototoxicity of the parent TMA was abolished, the photoadduct formation with DNA under UVA irradiation was investigated in the new TMA analogs by means of TLC analysis upon UVA irradiation of DNA/compound mixture.

Typically, if photoadducts between the lactone ring and thymine took place upon irradiation, the photoadducts appear as a violet fluorescent band at lower Rf values when observed under UV light, whereas the unchanged compounds (not photoreacted) appear at higher Rf values.

As shown in the Supplementary Materials, only TMA, used as a reference, showed photoadducts (Figure S2A) upon UVA irradiation, while the other compounds exhibited only the unchanged bands (Figure S2B–D, on **4-PhDMA**, **6-PhDMA**, and **pANDMA**, as representative compounds), thus demonstrating that they do not react with DNA under UVA irradiation.

Interestingly, the abolition of photoreaction is achieved by hindering positions 4 or 6, despite the distance of the latest one from the photoinducible double bonds on the furocoumarins ring. This is probably due to the non-planar shape of the 6-arylderivatives, preventing the intercalation into DNA and the subsequent addition.

#### 2.2.3. In Vitro Toxicity Studies: Evaluation of Mutagenic Effects (Ames Test)

To investigate whether changes in the structure of TMA abolish the mutagenic effect observed with this class of compounds, the most promising TMA analogs were evaluated by the Ames test. Compounds **4-PhDMA** and **pANDMA** were tested for genotoxicity evaluation using the histidine-requiring *Salmonella typhimurium* mutant TA97A, TA98, TA100, and TA1535 strains that allow checking frameshift mutation and base pair substitution, with and without metabolic activation induced by S9 Mix.

The histidine-requiring *Salmonella typhimurium* mutant strains described are particularly appropriate to check the genotoxic potential of new chemical compounds because they both have a plasmid that induces an increase in error-prone DNA damage repair (pKM101), and mutations that induce increased permeability to larger molecules through a defective lipopolysaccharide layer (*rfa* mutations) and avoid the possibility to repair excision DNA damage (*uvrB* mutations) [15,16].

The number of spontaneous revertants is relatively constant, but it dramatically increases—generally in a dose-dependent way—when a mutagen is added to the plate. In general, the cut-off to discriminate between a mutagenic and nonmutagenic result is considered as a two-fold revertants increase over the response of the negative control (spontaneous revertants).

For the above-reported considerations, the Ames test is generally considered an appropriate preliminary screening tool to determine the mutagenic potential of new chemicals for different industrial applications, from agrochemicals, pharmaceuticals, and medical devices to health products (e.g., for personal care, cosmetics, nutraceuticals, etc.). For the high significant predictive capacity, the regulatory agencies require the Ames test for the registration or acceptance of new compounds [16].

The results obtained for the compounds **4-PhDMA** and **pANDMA** are reported in the Supplementary Materials (Tables S1–S3): data were obtained employing the cited mutant, according to the method reported by Maron & Ames [15]. None of the samples had genotoxic activity at all concentrations tested with all the *Salmonella* strains. In conclusion, all of the samples can be considered non-genotoxic for the Ames test.

#### 2.2.4. In Vitro Toxicity Studies: Evaluation of the Cytotoxic Activity toward HepG2 Cells

Since the compounds are designed for oral administration and are likely to undergo the hepatic first-pass effect, we tested their cytotoxic potential toward HepG2 hepatoma cells. We could exclude the significant cytotoxicity of **4-PhDMA**, **pANDMA, and pANDMA mesylate,** as well as of the other compounds we screened, as cell viability was greater than 75% at the highest concentration tested (50 μM) (Figure S3 in the Supplementary Materials).

### 2.3. Pharmacokinetic Profile

The main pharmacokinetic parameters of **4-PhDMA**, **pANDMA**, and **pANDMA mesylate** were evaluated in c57BL/6 male mice, using water as dispersing vehicle. In addition, previously reported **IPEMA** was also tested, as it appeared to be the most active compound of all the synthesized TMA analogs.

**Figure 6:**
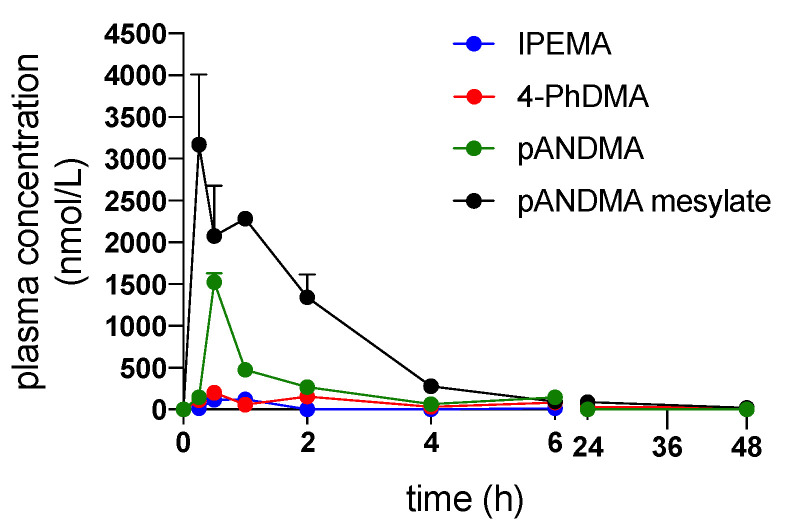
Plasma concentration versus time curves obtained after administration of the selected compounds **IPEMA**, **4-PhDMA**, **pANDMA**, and **pANDMA mesylate** suspended or dissolved in water to c57BL/6 male mice.

Figure 6 shows the plasma concentration versus time curves obtained after administration of the selected compounds.

The results highlight that the oral bioavailability of lipophilic TMA derivatives (**IPEMA**, **4-PhDMA,** and **pANDMA**) was very low, due to their poor water solubility, while the hydrophilic salt **pANDMA mesylate** was nearly four-times more bioavailable than **pANDMA** (Table 4).

#### 2.3.1. Formulation of Lipophilic TMA Analogs

Since the oral bioavailability of lipophilic molecules is limited due to their poor water solubility, the most promising lipophilic TMA derivatives (**4-PhDMA** and **IPEMA**) were formulated in self-emulsifying formulations (self-emulsifying drug delivery systems, SEDDS). SEDDS are mixtures containing drugs, an oil phase, surfactant, and co-surfactant/cosolvents that spontaneously self-emulsify upon contact with the aqueous environment in the gastrointestinal tract producing a milky or transparent emulsion able to improve drug solubilization. Furthermore, other phenomena, such as reduced efflux mediated by P-glycoproteins, along with improved lymphatic transport and intestinal wall permeability, can also contribute to an increase in oral bioavailability [17]. Thus, the most promising lipophilic derivatives of TMA (**4-PhDMA** and **IPEMA**) were formulated in two self-emulsifying formulations, previously built for the parent TMA [18], and characterized by the presence of two different oil phases. The first formulation, named A, contains a mixture of monoglycerides, diglycerides, and triglycerides of long-chain fatty acids, mainly linoleic and oleic acid (Maisine^®^ CC) as the oil phase, instead the second formulation, named B, contains a mixture of medium-chain triglycerides (mainly capric and caprylic acid derivatives (LabrafacTM Lipophile WL1349) as the oil phase.

The formulations were characterized by measuring the droplet size and PDI. The results are reported in Table 5.

The results show that both formulations are produced after dispersion emulsion with a droplet size smaller than 20 nm with a narrow droplet size distribution (PDI < 0.7); therefore, after oral administration, diffusion and lipid digestion processes can be facilitated.

#### 2.3.2. Bioavailability of IPEMA and 4-PhDMA Formulated in SEDDS

**Figure 7:**
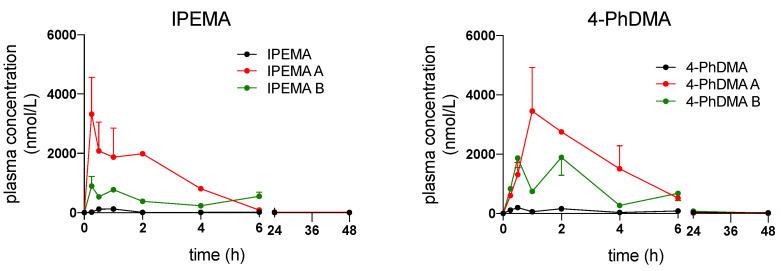
Plasma concentration versus time curves obtained after administration of the selected compounds **IPEMA** and **4-PhDMA**, suspended in water or formulated in SEDDS to c57BL/6 male mice.

Figure 7 shows the comparison between the plasma concentration versus time curves obtained after the administration of the selected lipophilic compounds **IPEMA** and **4-PhDMA** suspended in water and formulated in SEDDS.

The PK profiles clearly indicate that the SEDDS formulations were able to significantly increase the oral bioavailability of lipophilic derivatives.

In particular, the bioavailability of **IPEMA** formulated in SEDDS was increased 20-fold and 16-fold for A and B, respectively, while for **4-PhDMA** it was increased 6.4-fold and 4.8-fold for A and B, respectively (Table 6). Formulations A containing a mixture of long-chain mono, di, and triglycerides as oil phase were more effective than formulations B containing medium-chain triglycerides; this is probably due to the higher solvent capacity of the mixture of long-chain mono, di and triglycerides, in particular, after oral administration [19].

## 3. Materials and Methods

### 3.1. Chemistry

All the general and detailed synthetic procedures, characterization data, and mass spectra can be found in Supplementary Materials.

### 3.2. Biology

#### 3.2.1. CFTR Functional Rescue

The effect of TMA analogs on CFTR function was tested in CFBE41o- overexpressing F508del and the high sensitivity halide-sensing yellow fluorescent protein (HS-YFP) YFP-H148Q/I152L (a generous gift from N. Pedemonte, Gaslini Institute, Genova, Italy). YFP CF cells were seeded on round glass coverslips and preincubated for 48 h with vehicle or test compounds or VX809 or TMA. At the time of the assay, cells were washed in Dulbecco’s PBS (in mM: 137 NaCl, 2.7 KCl, 8.1 Na_2_HPO_4_, 1.5 KH_2_PO_4_, 1 CaCl_2_, and 0.5 MgCl_2_, pH 7.4) and subsequently incubated with a stimulation cocktail (20 μM forskolin and 5 μM VX770) in the presence and absence of 10 μM CFTR inhibitor, CFTRInh-172 (Sigma), for 30 min. The coverslips were then transferred on a Nikon TMD inverted microscope through a Nikon Fluor 40 objective. The signal was acquired with a Hamamatsu C2400–97 charge-coupled intensified video camera at a rate of 1 frame/3 s. The fluorescence coming from every single cell was analyzed by custom software (Spin, Vicenza, Italy). The results are presented as transformed data to obtain the percentage signal variation (F*x*) relative to the time of addition of the stimulus, according to the equation: F*x* = ([F*t* − F*o*]/F*o*) × 100, where F*t* and F*o* are the fluorescence values at time *t* and at the time of stimulus addition, respectively. The assay of each sample consists of a continuous 99-s fluorescence reading with 30 s before and 69 s after injection of the iodide-rich Dulbecco’s PBS (in mM: 137 NaI, 2.7 KCl, 8.1 Na_2_HPO_4_, 1.5 KH_2_PO_4_, 1 CaCl_2_, and 0.5 MgCl_2_, pH 7.4) to reach a final iodide concentration of 50 mM [20]. The different sensitivity of HS-YFP toward iodide and chloride allows assays to be performed by measuring the transport of anions through the plasma membrane as changes in cell fluorescence. For this assay, cells expressing HS-YFP are equilibrated in a physiological chloride-rich saline solution (e.g., Dulbecco’s PBS). During fluorescence reading, cells are exposed to a high concentration of iodide. Iodide influx quenches the cell fluorescence with a rate that depends on the permeability of the cell membrane to halides, and therefore, on the activity of the anion channels or transporters [21].

#### 3.2.2. Phototoxicity

UVA irradiation was carried out by using Philips HPW 125 lamps, mainly emitting at 365 nm. The total energy that struck the sample was monitored by means of a radiometer (Variocontrol, Waldmann, Villingen-Schwenningen, Germany), equipped with a Variocontrol UV Sensor (Waldmann). The radiant power emitted by the UVA lamp was about 8 mW/cm^2^. The samples were maintained at room temperature during irradiation.

The test compounds were dissolved in ethanol (2 mg/mL) and salmon testes DNA (Sigma-Aldrich) was dissolved in 10 mM NaCl and 1 mM EDTA solution (1.5 × 10^−3^ M). Each test solution (250 μL) was added dropwise to the DNA solution (5 mL). The mixture was irradiated with 15 J/cm2 UVA light in a glass dish for 1 h. After irradiation, the DNA was precipitated with 1 M NaCl and two volumes of cold ethanol; the precipitated DNA was collected, washed with 80% ethanol, dried, and then dissolved in water. To obtain hydrolysis, the final solution was acidified with 1 M HCl, heated at 100 °C for 1 h, neutralized with NaOH and extracted three times with chloroform. Then, all of the organic layers were collected, dried under a high vacuum, and dissolved in ethanol. The hydrolyzed mixtures were separated by thin layer chromatography (TLC; F_254_ plates, 0.25 mm, Merck, Darmstadt, Germany) eluting with EtOAc/EtOH (9:1). The plates were analyzed with a Mineral Light Lamp VL-4LC VILBERT-LOURMAT (Vilber, Marne-la-Vallée, France).

#### 3.2.3. Mutagenicity Assay

The mutagenicity assay was performed for the test compounds following the plate incorporation method with the histidine-requiring Salmonella typhimurium mutant TA97A, TA98, TA100, and TA1535 strains purchased by Molecular Toxicology Inc. (Boone, NC, USA; moltox.com). All strains (100 µL per plate of fresh overnight cultures) were checked with and without the addition of 0.5 mL of a 5% S9 exogenous metabolic activator (S9 mix). The lyophilized post-mitochondrial supernatant S9 mix (Aroclor 1254-induced, Sprague–Dawley male rat liver in 0.154 M KCl solution), commonly used for the activation of pro-mutagens in mutagenic metabolites (Molecular Toxicology, Inc., Boone, NC, USA), was stored at −80 °C before use. The concentration tested for all the samples were 5, 10, 20, 50, and 100 µL/plate from a stock solution of 500 nM. A fully grown culture of the appropriate tester strain (0.1 mL) was added to 2 mL molten top agar (0.6% agar, 0.6% NaCl, 0.5 mM L-histidine/biotin solution) at 46 °C, together with 0.1 mL of each sample solution at different concentrations, and 0.5 mL S9 mix for assays with metabolic activation. The ingredients were thoroughly mixed and poured onto minimal glucose agar plates (1.5% agar in 2% Vogel–Bonner medium E with 5% glucose solution). DMSO was used as negative a control (100 µL/plate). Positive controls were prepared as follows: 2-aminoanthracene (2 µg/plate) and 2-nitrofluorene (2 µg/plate) for TA 97A, TA98 and TA1535 with and without metabolic activator (S9 mix), respectively; 2-aminoanthracene (2 µg/plate) and sodium azide (2 µg/plate) for TA100, with and without metabolic activator (S9 mix), respectively. The plates were incubated at 37 °C for 72 h and then his+ revertants were checked and counted using a Colony Counter 560 Suntex (Antibioticos, Italy). A sample was considered mutagenic when the observed number of colonies was at least twofold over the spontaneous level of revertants [15,16]. All determinations were made in triplicate. Statistical analysis: relative standard deviations were calculated using the statistical software STATISTICA 6.0 (StatSoft, Italia srl, Vigonza, Italy).

#### 3.2.4. Cytotoxicity Assay

The potential toxicity of TMA analogs on hepatic tissue was assessed by evaluating the viability of HepG2 cells (ATCC, HB-8065) using the MTT assay, as previously reported [22]. Cells were maintained in DMEM complete medium (10% FBS, 1% l-glutamine and 1% PEN-STREP) and seeded in a 96-well plate (5 × 10^4^ cells/mL). They were treated for 24 h with TMA analogs (concentration range from 3.125 μM to 50 μM). At the end of the treatment, cells were washed and an MTT solution (0.5 mg/mL in complete medium) was added to each well and incubated for 4 h at 37 °C. The solution was discarded, and the produced blue formazan crystals were dissolved with acidified isopropanol (2% HCl). Absorbance was measured at 570 nm using a multiplate reader (Victor Nivo, Perkin Elmer). The percentage of viable cells was calculated considering untreated cells as control.

### 3.3. Formulation of Lipophilic TMA Derivatives

Self-emulsifying formulations (SEDDS) were prepared as described in previous work [18], in particular, medium-chain triglycerides (LabrafacTM Lipophile WL1349) or glycerol/glyceryl monolinoleate (Maisine^®^ CC) were selected as the oil phase, diethylene glycol-monoethyl ether (Transcutol^®^ HP) as the cosolvent, and polyoxyl-35-castor oil (Cremophor^®^ EL) as the surfactant. Ten percent (*w*/*w*) of the oil phase, 40% (*w/w*) of cosolvent and 50% (*w/w*) of surfactant were well mixed, then the TMA derivative (4-PhDMA or IPEMA) was introduced to obtain formulations with a drug concentration of 4 µg/µL. Loaded formulations were characterized by measuring the droplet size, and the polydispersity index (PDI), using a laser light scattering analyzer (Zetasizer ZS 90, Malvern Instruments, Worcestershire, UK). For the measurement, SEDDS were diluted in a ratio of 1:250 (*v/v*) with distilled water and mixed for 1 min prior to testing. From the light scattering signal (monitored at 25 °C and 173° in automatic mode), the intensity-weighed diameter of dispersion droplets (reported as the z-average) and the PDI were calculated using the manufacturer’s software

### 3.4. Pharmacokinetic Profile

#### 3.4.1. In Vivo Pharmacokinetics

All experimental procedures involving animals were conducted in accordance with the 3R principle to minimize their pain and discomfort. The experimental design and the procedures have been authorized by the Italian Ministry of Health (Authorization no. 853-2018-PR obtained on 11 November 2018). All mice were maintained in IVC cages with free access to food and drink with a 12/12 h of dark/light. For the PK analysis, C57Bl6 male mice (n = 3 per time point) were gavaged with selected compounds (20 mg/kg), suspended in water, or dissolved in SEDDS formulations, after an overnight fast. Plasma samples were collected from the submandibular plexus before and 0.5, 1, 2, 3, 4, 6 24 and 48 h after the administration of the compounds or their formulations. The main PK parameters have been calculated with standard formulas using the PK Solver plug-in for Microsoft Excel and analyzed by means of Student’s t-test or one-way ANOVA using GraphPad Prism ver. 8.0 software (GraphPad Software Inc., San Diego, CA, USA), when appropriate. If not otherwise stated, the PK data are expressed as mean ± SD. *p* < 0.05 was considered statistically significant.

#### 3.4.2. Quantification of TMA Analogs in Plasma Samples by UHPLC-HRMS

The TMA analogs were extracted and analyzed from mouse plasma as already reported [18]. Briefly, 100 µL of plasma were treated with 300 µL of cold acetonitrile spiked with 80 nM of internal standard (IS, 6,4′-dimethylangelicin) [23], and then the samples were centrifuged for 10 min at 12,000× *g* (Hettich Mikro 120 Benchtop Centrifuge) and 25 °C. Ten microliters of the supernatant were injected into the UHPLC system.

The UHPLC-HRMS system was equipped with an Agilent 1260 Infinity II LC liquid chromatographer coupled to an Agilent 6545 LC/Q-TOF mass analyzer (Agilent Technologies, Palo Alto, CA, USA). The analytical column was a Kinetex 2.6 μm C18 Polar, 100 A, 100 × 2.1 mm (Phenomenex, Bologna, Italy), thermostated at 30 °C. The mobile phase components A and B were water and methanol, respectively, both containing 10 mM ammonium formate. The eluent flow rate was 0.25 mL/min. The mobile phase gradient profile was as follows (t in min): t0-3 2% B; t3-18 2-100% B, t18-20 100% B; t20-30 0% B. The MS conditions were: electrospray (ESI) ionization in positive mode, Gas Temp 325 °C, Drying Gas 10 L/min, Nebulizer 20 psi, Sheath Gas Temp 400 °C, Sheath Gas Flow 12 L/min, VCap 4000 V, Nozzle Voltage 0 V, Fragmentor 180 V. Centroid full scan mass spectra were recorded in the range 100–1000 m/z with a scan rate of 1 spectrum/s. MS data were analyzed using the Mass Hunter Qualitative Analysis software (Agilent Technologies, Palo Alto, CA, USA).

Chromatographic peaks of the TMA analog were identified and integrated by considering the Extracted Ion Chromatogram (EIC) of the [M+H]^+^ species selected with a window of 5 ppm and normalized by the IS. Quantification of TMA analogs was carried out by external calibration of normalized signals using a seven-point calibration curve obtained by spiking blank plasma with each TMA analog in the range of 20–3000 nM. Linearity showed an R2 > 0.98 and the limit of detection of the method was assessed to be approximately 10 nM.

## 4. Conclusions

A small library of TMA analogs was designed and synthesized to identify new agents with improved safety profiles and bioavailability with respect to the parent TMA. From preliminary screening data on CFTR correction activity, together with previously obtained data on **IPEMA** [11], we try to outline the structural determinants that allow maintaining the correction potential. A certain steric hindrance at either positions 4 or 6 is essential to abolish photoreactivity towards DNA. However, the simultaneous presence of bulky substituents in both positions compromised the maximal CFTR functional rescue achieved. A balanced combination was found to be the presence of a phenyl group at positions 4 or 6, and a methyl substituent at positions 6 or 4, respectively, as exhibited by **4-PhDMA** and **p-ANDMA**, the most active derivatives of the series. Only small alkyl groups are tolerated together at the two positions, as is the case for **IPEMA**. In the furan ring, the presence of a methyl group at position 4′ leads to a better in vitro pharmacological profile compared to the unsubstituted derivatives. All of the analogs were demonstrated to be nongenotoxic for the Ames test.

In terms of pharmacokinetic profile, the more lipophilic compounds showed poor bioavailability and should be opportunely formulated as SEDDS. On the other hand, the introduction of a hydrophilic group in the *para* position of the 6-phenyl substituent gives rise not only to an improvement in bioavailability but also to the opportunity to make a soluble salt that demonstrated appropriate C_max_ and AUC, together with low interindividual variability in absorption.

In conclusion, by modifying the parent TMA at position 4 or 6, we obtained a large series of analogs with an improved safety profile and bioavailability, per se or after incorporation into suitable delivery systems. Furthermore, a CFTR functional screening assay selected some of these analogs to undergo future extensive electrophysiological experiments in CF primary bronchial epithelial cells, to evaluate their ability to functionally rescue the F508del CFTR protein.

## Figures and Tables

**Table 1:** R substituents for the A series of TMA analogs.

Compounds	R_4_	R_6_	ID Final Product ^1^
**1**	-	Me	-
**2**	-	Et	-
**3, 8, 11**	*c*Pr	Me	**CPDMA**
**4, 9, 12**	Ph	Me	**4-PhDMA**
**5, 10, 13**	Ph	Et	**4-PhEMA**
**3, 14, 17**	*c*Pr	Me	**CPMA**
**6, 15, 18**	*i*Pr	Me	**IPMA**
**7, 16, 19**	*i*Pr	Et	**IPEA**

^1^ The ID is derived from the chemical name for TMA: CP, cyclopropyl; DMA, dimethylangelicin; Ph, phenyl; EMA, ethylmethylangelicin; MA, methylangelicin; IP, isopropyl; EA, ethylangelicin.

**Table 2:** R substituents for the B series of TMA analogs.

Compounds	R_4_	R_6_	ID Final Product ^1^
**24**	Me	Ph	**6-PhDMA**
**25**	Me	*p*-NH_2_Ph	**pANDMA**
**26**	Me	*m*-NH_2_Ph	**mANDMA**
**27**	Me	Pyridyl	**PyDMA**
**28**	Me	Thienyl	**ThiDMA**

^1^ The ID is derived from the chemical name as for TMA: Ph, phenyl; DMA, dimethylangelicin; pAN, para-aniline; mAN, meta-aniline; Py, pyridyl; Thi, thienyl.

**Table 3:** R substituents for the three series of TMA analogs.

Compounds	R_4_	R_6_	ID Final Product ^1^
**29**	-	Ph	-
**30, 32, 34**	*i*Pr	Ph	**IPPhMA**
**31, 33, 35**	*c*Pr	Ph	**CPPhMA**

^1^ The ID is derived from the chemical name for TMA: IP, isopropyl; Ph, phenyl; MA, methylangelicin; CP, cyclopropyl.

**Table 4:** Main PK parameters of the selected compounds.

Compound	t_1/2_ (h)	C_max_ (nmol/L)	T_max_ (h)	AUC 0-inf (nmol/L*h)	CL/F (mL/h/g)
**IPEMA**	9.01 ± 1.34	120.4 ± 55.3	1 ± 0	394.50 ± 98.72	0.0633 ± 0.005
**4-PhDMA**	21.13 ± 0.98	221.77 ± 184.09	1.25 ± 1.06	2747.36 ± 513.61	0.0014 ± 0.0014
**pANDMA**	1.91 ± 0.71	1523.5 ± 260.9	0.5 ± 0	2405.4 ± 208.2	0.0083 ± 0.0007
**pANDMA mesylate**	1.87 ± 0.43	3868.3 ± 1065.2	0.375 ± 0.177	9402.6 ± 742.2	0.0091 ± 0.0002

The experiment was performed in c57BL/6 male mice. t_1/2_: elimination half-life; C_max_: maximum plasma concentration, T_max_: time to maximum plasma concentration, AUC0-inf: area under the plasma concentration vs. time curve, CL/F: apparent oral clearance.

**Table 5:** Properties of the SEDDS developed for **IPEMA** and **4-PhDMA**.

Compound	Formulation	Droplet Size (nm)	PDI (-)
**IPEMA**	A	14.62 ± 2.80	0.24
**IPEMA**	B	15.10 ± 1.50	0.18
**4-PhDMA**	A	13.23 ± 2.10	0.31
**4-PhDMA**	B	14.56 ± 1.57	0.35

**Table 6:** Main PK parameters of the selected compounds formulated in SEDDS.

Compound/. Formulation	t_1/2_ (h)	C_max_ (nmol/L)	T_max_ (h)	AUC 0-inf (nmol/L*h)	CL/F (mL/h/g)
**IPEMA A**	5.99 ± 0.87	3184,582 ± 897,45	0.25 ± 0	12,456.1 ± 1456.3	0.0021 ± 0.007
**IPEMA B**	6.15 ± 1.09	845.73 ± 76.54	0.25 ± 0	6671.16 ± 432.13	0.0037 ± 0.002
**4-PhDMA A**	6.08 ± 4.78	4374.8 ± 2296.6	1.5 ± 0.71	17,699.4 ± 6170.2	0.0012 ± 0.0005
**4-PhDMA B**	7.31 ± 4.29	2683.0 ± 370.4	1.25 ± 1.06	13,337.8 ± 6463.4	0.0017 ± 0.0008

The experiment was performed in c57BL/6 male mice. t_1/2_: elimination half-life; C_max_: maximum plasma concentration, T_max_: time to maximum plasma concentration, AUC0-inf: area under the plasma concentration vs. time curve, CL/F: apparent oral clearance.

## Data Availability

The data presented in this study are available in this article and in the Supplementary Materials.

## Supplementary Materials

The following supporting information can be downloaded at: https://www.mdpi.com/article/10.3390/ijms231911528/s1.
