# Supplementary material for: Improved Trimethylangelicin Analogs for Cystic Fibrosis: Design, Synthesis and Preliminary Screening
Source: Int J Mol Sci. 2022 Sep 29;23(19):11528. doi: 10.3390/ijms231911528 (PMC9570109; doi:10.3390/ijms231911528)
Supplement: Supplementary file 1 [file ijms-23-11528-s001.zip › ijms-1937858-supplementary.pdf]

Supplementary Materials

Improved trimethylangelicin analogs for cystic fibrosis: design, synthesis and preliminary screening

Christian Vaccarin[1,2,#], Daniela Gabbia[1,#], Erica Franceschinis[1], Sara De Martin[1], Marco Roverso[3], Sara Bogialli[3], Gianni Sacchetti[4], Chiara Tupini[4], Ilaria Lampronti[4,5], Roberto Gambari[4,5], Giulio Cabrini[4,5], Maria Cristina Dechecchi[6], Anna Tamanini[6], Giovanni Marzaro[1], and Adriana Chilin[1,5,*]

[1]Department of Pharmaceutical and Pharmacological Sciences, University of Padova, via Marzolo 5, 35131 Padova (Italy)

[2]Center for Radiopharmaceutical Sciences ETH-PSI-USZ, Paul Scherrer Institute, 5232 Villigen-PSI (Switzerland)
[3]Department of Chemical Sciences, University of Padova, via Marzolo 1, 35131 Padova (Italy)
[4]Department of Life Sciences and Biotechnology, University of Ferrara, Via Fossato di Mortara 74, 44121 Ferrara, Italy

[5]Center of Innovative Therapies for Cystic Fibrosis (InnThera4CF), University of Ferrara, Via Fossato di Mortara 74, 44121 Ferrara, Italy

[6]Department of Neurosciences, Biomedicine and Movement, Section of Clinical Biochemistry, University of Verona, Piazzale Stefani 1, 37126 Verona, Italy

[#]These authors contributed equally to this work
*****Correspondence: adriana.chilin@unipd.it

Corresponding author: Adriana Chilin (adriana.chilin@unipd.it)

CHEMISTRY – MATERIALS AND METHODS **pg. 2**

MASS SPECTRA OF THE FINAL COMPOUNDS **pg. 10**

EFFECT OF FINAL COMPOUNDS ON CFTR POTENTIATION **pg. 15**

PHOTOTOXICITY **pg. 16**

MUTAGENICITY ASSAY ON *SALMONELLA TYPHIMRIUM* STRAINS **pg. 17**

CYTOTOXICITY ASSAY ON HepG2 CELLS **pg. 18**

CHEMISTRY

MATERIALS AND METHODS

General Information. All commercial chemicals and solvents (Carlo Erba, Merck) used were analytical grade and were used without further purification. Microwave-assisted reactions were performed on a CEM Discover monomode reactor in closed devices with the temperature monitored by a built-in infrared sensor and the automatic control of the power. Melting points (uncorrected) were determined using a Gallenkamp MFB-595-010M capillary melting point apparatus. Analytical thin layer chromatography (tlc) was performed on pre-coated silica gel plates (Merck 60-F-254, 0.25 mm), which were developed on a mixture of $CHCl_3$/MeOH (9/1). The NMR spectra were recorded on a Bruker 300-AMX spectrometer with TMS as internal standard. Coupling constants are given in Hz. HRMS spectra were acquired using a XEVO G2-S Qtof (Waters) mass spectrometer with direct injection of the sample and collecting data in positive ion mode. The purity of compounds was determined by elemental analysis. Elemental analyses were performed on a Perkin-Elmer 2400 analyser and all values were within ± 0.4 % of the theoretical values.

Synthesis of 7-hydroxybenzopyran-2-ones 3-7 (A series).

General procedure. To a solution of **1** or **2** (10.0 mmol) in the appropriate acetoacetic ester (10.0 mmol), H_2SO_4 (7 mL) was added dropwise. The resulting homogeneous mixture was stirred at room temperature for 1 h, and then was poured into an ice/water mixture (100 g). The obtained precipitate was filtered and washed with water to give the corresponding 7-hydroxybenzopyran-2-ones **3-7**.

4-Cyclopropyl-7-hydroxy-6-methylbenzopyran-2-one (3). From **1**: yield 83%: mp 162 °C. ^{1}H-NMR ($CDCl_3$): 7.63 (s, 1 H, 5-H); 6.99 (s, 1 H, 8-H); 5.86 (s, 1 H, 3-H); 2.33 (s, 3 H, CH_3); 2.12-2.04 (m, 1 H, cyclopropyl-CH); 1.17-1.11 (m, 2 H, cyclopropyl-CH_2); 0.87-0.81 (m, 2 H, cyclopropyl-CH_2).

7-Hydroxy-6-methyl-4-phenylbenzopyran-2-one (4). From **1**: yield 87%: mp 265 °C. ^{1}H-NMR ($CDCl_3$): 7.56-7.50 (m, 3 H, 3'-H, 4'-H and 5'-H); 7.47-7.41 (m, 2 H, 2'-H, 6'-H); 7.20 (s, 1 H, 5-H); 7.00 (s, 1 H, 8-H); 6.20 (s, 1 H, 3-H); 2.21 (s, 3 H, 6-CH_3).

6-Ethyl-7-hydroxy-4-phenylbenzopyran-2-one (5). From **2**: yield 95%: mp 238 °C. ^{1}H-NMR ($CDCl_3$): 7.56-7.50 (m, 3 H, 3'-H, 4'-H, 5'-H); 7.49-7.43 (m, 2 H, 2'-H, 6'-H); 7.22 (s, 1 H, 5-H); 7.02 (s, 1 H, 8-H); 6.21 (s, 1 H, 3-H); 2.61 (q, J=7.3, 2 H, $C\underline{H}_2CH_3$); 1.16 (t, J=7.4, 3 H, $CH_2C\underline{H}_3$).

7-Hydroxy-4-isopropyl-6-methylbenzopyran-2-one (6). From **1**: yield 40%: mp 147 °C. ^{1}H-NMR ($CDCl_3$): 7.41 (s, 1H, 5-H); 6.96 (s, 1H, 8-H); 6.17 (d, *J* = 1.1 Hz, 1H, 3-H); 3.27 (sept/d, *J* = 7.2 H, 1.1 Hz, 1H, $C\underline{H}(CH_3)_2$); 2.31 (s, 3H, 6-Me); 1.31 (d, *J* = 7.2 Hz, 6H, $CH(C\underline{H}_3)_2$).

6-Ethyl-7-hydroxy-4-isopropylbenzopyran-2-one (7). From **2**: yield 52%: mp 168 °C. ^{1}H-NMR ($CDCl_3$): 7.41 (s, 1H, 5-H); 7.05 (s, 1H, 8-H); 6.17 (d, *J* = 1.1 Hz, 1H, 3-H); 3.30 (sept/d, *J* = 7.2 H,

1.1 Hz, 1H, $C\underline{H}(CH_3)_2$); 2.71 (q, *J* = 7.5 Hz, 2H, $C\underline{H}_2CH_3$); 1.51 (d, *J* = 7.2 Hz, 6H, $CH(C\underline{H}_3)_2$); 1.26 (t, *J* = 7.5 Hz, 3H, $CH_2C\underline{H}_3$).

Synthesis of 7-O-(2’-oxopropyl)ethers 8-10 (A series).

General procedure. A mixture of 7-hydroxybenzopyran-2-ones **3-5** (5.0 mmol), chloroacetone (0.5 mL, 6.2 mmol), KI (10 mg), and anhydrous K_2CO_3 (2.1 g, 15.0 mmol) in acetone (75 mL) was refluxed for 12 h. After cooling, the solid was filtered off and washed with fresh acetone (25 mL). The solvent was evaporated from the combined filtrate and washings to give the corresponding 7-(2’-oxopropyloxy)benzopyran-2-ones **8-10**.

4-Cyclopropyl-6-methyl-7-(2’-oxopropyloxy)benzopyran-2-one (8). From **3**: yield 87%: mp 120 °C. ^{1}H-NMR ($CDCl_3$): 7.67 (s, 1 H, 5-H); 6.59 (s, 1 H, 8-H); 5.89 (s, 1 H, 3-H); 4.62 (s, 2 H, $C\underline{H}_2COCH_3$); 2.37 (s, 3 H, $CH_2COC\underline{H}_3$); 2.33 (s, 3 H, 6-CH_3); 2.11-2.03 (m, 1 H, cyclopropyl-CH); 1.17-1.11 (m, 2 H, cyclopropyl-CH_2); 0.87-0.81 (m, 2 H, cyclopropyl-CH_2).

6-Methyl-7-(2’-oxopropyloxy)-4-phenylbenzopyran-2-one (9). From **4**: yield 95%: mp 151 °C. ^{1}H-NMR ($CDCl_3$): 7.56-7.50 (m, 3 H, 3"-H, 4"-H, 5"-H); 7.47-7.41 (m, 2 H, 2"-H, 6"-H); 7.24 (s, 1 H, 5-H); 6.66 (s, 1 H, 8-H); 6.22 (s, 1 H, 3-H); 4.65 (s, 2 H, $C\underline{H}_2COCH_3$); 2.34 (s, 3 H, $CH_2COC\underline{H}_3$); 2.26 (s, 3 H, 6-CH_3).

6-Ethyl-7-(2’-oxopropyloxy)-4-phenylbenzopyran-2-one (10). From **5**: yield 40%: mp 145 °C. ^{1}H-NMR (DMSO-d_6): 7.61-7.51 (m, 5 H, 3"-H, 4"-H, 5"-H, 2"-H, 6"-H); 7.19 (s, 1 H, 5-H); 7.03 (s, 1 H, 8-H); 6.21 (s, 1 H, 3-H); 5.04 (s, 2 H, $C\underline{H}_2COCH_3$); 2.58 (q, J=7.5, 2 H, $C\underline{H}_2CH_3$); 2.20 (s, 3 H, $CH_2COC\underline{H}_3$); 1.08 (t, J=7.5, 3 H, $CH_2C\underline{H}_3$).

Synthesis of 2*H*-furo[2,3-*h*]-1-benzopyran-2-ones 11-13 (A series).

General procedure. A 4% ethanolic potassium hydroxide solution (15 mL) was slowly added to a solution of 7-(2’-oxopropyloxy)benzopyran-2-ones **8-10** (1.0 mmol) in absolute ethanol (50 mL), and the mixture was refluxed in the dark for 1 h. The solution was cooled to room temperature, diluted with water (50 mL) and acidified with diluted HCl. The ethanol was removed by evaporation under reduced pressure and the residue was extracted with $CHCl_3$ (3 x 50 mL). The organic phase was evaporated under reduced pressure and the solid residue was purified by column chromatography (eluent: CE/EtOAc, 8:2) to give the corresponding 2*H*-furo[2,3-*h*]-1-benzopyran-2-ones **11-13**.

4-Cyclopropyl-6,9-dimethyl-2H-furo[2,3-h]-1-benzopyran-2-one (11, CPDMA). From **8**: yield 33%: mp 155 °C. ^{1}H-NMR ($CDCl_3$): 7.56 (s, 1 H, 5-H); 7.41 (q, J=1.4, 1 H, 5'-H); 5.99 (s, 1 H, 3-H); 2.54 (s, 3 H, 6-CH_3); 2.51 (d, J=1.1, 3 H, 4'-CH_3); 2.19-2.11 (m, 1 H, cyclopropyl-CH); 1.19-1.13 (m,

2 H, cyclopropyl-CH_2); 0.88-0.82 (m, 2 H, cyclopropyl-CH_2). HRMS (ESI-TOF) for $C_{16}H_{15}O_3$ $[M + H]^+$: calcd.: 255.1021, found: 255.0773. Anal. calcd. for $C_{16}H_{14}O_3$: C, 75.57; H 5.55; found: C, 75.60; H, 5.51.

6,9-Dimethyl-4-phenyl-2H-furo[2,3-h]-1-benzopyran-2-one (12, 4-PhDMA). From **9**: yield 20%: mp 190 °C. ^{1}H-NMR ($CDCl_3$): 7.56-7.50 (m, 3 H, 3'-H, 4'-H, 5'-H); 7.47-7.41 (m, 3 H, 2'-H, 6'-H, 5-H); 7.09 (q, J=1.3, 1 H, 8-H); 6.30 (s, 1 H, 3-H); 2.57 (d, J=1.3, 3 H, 9-CH_3); 2.43 (d, J=0.7, 3 H, 6-CH_3). HRMS (ESI-TOF) for $C_{19}H_{15}O_3$ $[M + H]^+$: calcd.: 291.1021, found: 291.0501. Anal. calcd. for $C_{19}H_{14}O_3$: C, 78.61; H 4.86; found: C, 78.63; H, 4.75.

6-Ethyl-9-methyl-4-phenyl-2H-furo[2,3-h]-1-benzopyran-2-one (13, 4-PhEMA). From **10**: yield 10%: mp 185 °C. ^{1}H-NMR ($CDCl_3$): 7.57-7.51 (m, 3 H, 3'-H, 4'-H, 5'-H); 7.50-7.43 (m, 3 H, 2'-H, 6'-H e 5-H); 7.12 (q, J=1.3, 1 H, 8-H); 6.30 (s, 1 H, 3-H); 2.84 (q, J=7.5, 2 H, $C\underline{H}_2CH_3$); 2.57 (d, J=1.3, 3 H, 9-CH_3); 1.25 (t, J=7.5, 3 H, $CH_2C\underline{H}_3$). HRMS (ESI-TOF) for $C_{20}H_{17}O_3$ $[M + H]^+$: calcd.: 305.1178, found: 305.0511. Anal. calcd. for $C_{20}H_{16}O_3$: C, 78.93; H 5.30; found: C, 78.81; H, 5.27.

Synthesis of 7-(2’,2’-diethoxyethyl)ethers 14-16 (A series).

General procedure. A mixture of alkyl-7-hydroxybenzopyran-2-ones **3**, **6** or **7** (5.0 mmol), chloroacetaldehyde diethylacetale (1.0 mL, 6.6 mmol), KI (10 mg), and anhydrous K_2CO_3 (2.8 g, 20.0 mmol) in DMF (25 mL) was refluxed for 18 h. After cooling, the mixture was poured into an ice/water mixture (100 g) and the obtained precipitate was collected by filtration. The residue was purified by column chromatography (eluent: $CHCl_3$) to give the corresponding alkyl-7-(2’,2’-diethoxyethoxy)-2*H*-1-benzopyran-2-ones **14-16**.

4-Cyclopropyl-7-(2’,2’-diethoxyethoxy)-6-methylbenzopyran-2-one (14). From **3**: yield 47%: mp 110 °C. ^{1}H-NMR ($CDCl_3$): 7.62 (s, 1 H, 5-H); 6.75 (s, 1 H, 8-H); 5.87 (s, 1 H, 3-H); 4.89 (t, J=5.1, 1 H, $CH_2C\underline{H}(OCH_2CH_3)_2$); 4.05 (d, J=5.3, 2 H, $C\underline{H}_2CH(OCH_2CH_3)_2$); 3.85-3.60 (m, 4 H, $CH_2CH(OC\underline{H}_2CH_3)_2$); 2.29 (s, 3 H, 6-$CH_3$); 2.12-2.01 (m, 1 H, cyclopropyl-CH); 1.23-1.29 (m, 6 H, $CH_2CH(OCH_2C\underline{H}_3)_2$); 1.16-1.09 (m, 2H, cyclopropyl-CH_2); 0.86-0.79 (m, 2 H, cyclopropyl-CH_2).

7-(2’,2’-Diethoxyethoxy)-4-isopropyl-6-methylbenzopyran-2-one (15). From **6**: yield 36%: mp 61 °C. ^{1}H-NMR ($CDCl_3$): 7.39 (s, 1 H, 5-H); 6.77 (s, 1 H, 8-H); 6.16 (s, 1 H, 3-H); 4.89 (t, J=5.0, 1 H, $CH_2C\underline{H}(OCH_2CH_3)_2$); 4.05 (d, J=5.1, 2 H, $C\underline{H}_2CH(OCH_2CH_3)_2$); 3.85-3.60 (m, 4 H, $CH_2CH(OC\underline{H}_2CH_3)_2$); 3.32-3.19 (m, 1 H, $C\underline{H}(CH_3)_2$); 2.27 (s, 3 H, 6-CH_3); 1.30 (d, J=6.8, 6 H, $CH(C\underline{H}_3)_2$); 1.26 (t, J=7.0, 6 H, $CH_2CH(OCH_2C\underline{H}_3)_2$).

7-(2’,2’-Diethoxyethoxy)-6-ethyl-4-isopropylbenzopyran-2-one (16). From **7**: yield 83%: mp 55 °C. ^{1}H-NMR ($CDCl_3$): 7.39 (s, 1 H, 5-H); 6.77 (s, 1 H, 8-H); 6.16 (s, 1 H, 3-H); 4.89 (t, J=4.9, 1 H, $CH_2C\underline{H}(OCH_2CH_3)_2$); 4.05 (d, J=5.4, 2 H, $C\underline{H}_2CH(OCH_2CH_3)_2$); 3.85-3.60 (m, 4 H,

$CH_2CH(OC\underline{H}_2CH_3)_2$); 3.35-3.20 (m, 1 H, $C\underline{H}(CH_3)_2$); 2.69 (q, J=7.3, 2H, $C\underline{H}_2CH_3$); 1.31 (d, J=6.8, 6 H, $CH(C\underline{H}_3)_2$); 1.26 (t, J=7.0, 6 H, $CH_2CH(OCH_2C\underline{H}_3)_2$); 1.22 (t, J=7.3, 3 H, $CH_2C\underline{H}_3$).

Synthesis of 2*H*-furo[2,3-*h*]-1-benzopyran-2-ones 17-19 (A series).

General procedure. A solution of alkyl-7-(2',2'-ethoxyethoxy)benzopyran-2-ones **14-16** (1.0 mmol) in TFA (7.5 mL) was microwave irradiated at 120 °C (power set point 200W; ramp time 1 minute; hold time 5 minutes). After cooling, the mixture was poured into an ice/water mixture (100 g) and the obtained precipitate was collected by filtration. The residue was purified by column chromatography (eluent: $CHCl_3$) to give the corresponding 2*H*-furo[2,3-*h*]-1-benzopyran-2-ones **17-19**.

4-Cyclopropyl-6-methyl-2H-furo[2,3-h]-1-benzopyran-2-one (17, CPMA). From **14**: yield 29%: mp 220 °C. ^{1}H-NMR ($CDCl_3$): 7.68 (d, J=2.2, 1 H, 5'-H); 7.62 (s, 1 H, 5-H); 7.13 (d, J=2.2, 1 H, 4'-H) 6.02 (s, 1 H, 3-H); 2.59 (s, 3 H, 6-CH_3); 2.22-2.12 (m, 1 H, cyclopropyl-CH); 1.21-1.14 (m, 2 H, cyclopropyl-CH_2); 0.90-0.84 (m, 2 H, cyclopropyl-CH_2). HRMS (ESI-TOF) for $C_{15}H_{13}O_3$ $[M + H]^+$: calcd.: 241.0865, found: 241.0294. Anal. calcd. for $C_{15}H_{12}O_3$: C, 74.99; H 5.03; found: C, 75.02; H, 4.99.

4-Isopropyl-6-methyl-2H-furo[2,3-h]-1-benzopyran-2-one (18, IPMA). From **15**: yield 28%: mp 135 °C. ^{1}H-NMR ($CDCl_3$): 7.68 (d, J=2.2, 1 H, 5'-H); 7.37 (s, 1 H, 5-H); 7.14 (d, J=2.2, 1 H, 4'-H) 6.29 (s, 1 H, 3-H); 3.44-3.29 (m, 1 H, $C\underline{H}(CH_3)_2$); 2.58 (s, 3 H, 6-CH_3); 1.35 (d, J=6.8, 6 H, $CH(C\underline{H}_3)_2$). HRMS (ESI-TOF) for $C_{15}H_{15}O_3$ $[M + H]^+$: calcd.: 243.1021, found: 243.0550. Anal. calcd. for $C_{15}H_{14}O_3$: C, 74.36; H 5.82; found: C, 74.40; H, 5.84.

6-Ethyl-4-isopropyl-2H-furo[2,3-h]-1-benzopyran-2-one (19, IPEA). From **16**: yield 24%: mp 110 °C. ^{1}H-NMR ($CDCl_3$): 7.68 (d, J=2.2, 1 H, 5'-H); 7.38 (s, 1 H, 5-H); 7.14 (d, J=2.2, 1 H, 4'-H) 6.29 (s, 1 H, 3-H); 3.46-3.31 (m, 1 H, $C\underline{H}(CH_3)_2$); 2.98 (q, J=7.5, 2H, $C\underline{H}_2CH_3$); 1.37 (t, J=7.5, 3 H, $CH_2C\underline{H}_3$); 1.22 (d, J=6.8, 6 H, $CH(C\underline{H}_3)_2$). HRMS (ESI-TOF) for $C_{16}H_{17}O_3$ $[M + H]^+$: calcd.: 257.1178, found: 257.0570. Anal. calcd. for $C_{16}H_{16}O_3$: C, 74.98; H 6.29; found: C, 75.96; H, 6.32.

Synthesis of 6-aryl-2*H*-furo[2,3-*h*]-1-benzopyran-2-ones 24-28 (B series).

6-Bromo-7-hydroxy-4-methylbenzopyran-2-one (21). To an ice-cold solution of 4-bromoresorcinol **20** (1.9 g, 10.0 mmol) in ethyl acetoacetate (1.3 mL, 10.0 mmol), H_2SO_4 (7 mL) was added dropwise and the mixture was stirred at room temperature for 1 h. The solution was poured into an ice/water mixture (100 g) and the obtained precipitate was filtered and washed with water to give **21** (2.1 g, yield 83%); mp 273 °C. ^{1}H-NMR ($CDCl_3$): 7.62 (s, 1 H, 5-H); 6.83 (s, 1 H, 8-H); 6.07 (q, J=1.1, 1 H, 3-H); 2.34 (d, J=1.1, 3 H, CH_3).

6-Bromo-4-methyl-7-(2'-oxopropyloxy)benzopyran-2-one (22). A mixture of **21** (1.3 g, 5.0 mmol), chloroacetone (0.5 mL, 6.2 mmol), KI (10 mg), and anhydrous K_2CO_3 (2.1 g, 15.0 mmol) in acetone (75 mL) was refluxed for 12 h. After cooling, the solid was filtered off and washed with fresh acetone (25 mL). The solvent was evaporated from the combined filtrate and washings to give **22** (1.1 g, yield 69%); mp 193°C. ^{1}H-NMR ($CDCl_3$): 7.80 (s, 1 H, 5-H); 6.67 (s, 1 H, 8-H); 6.20 (q, J=1.1, 1 H, 3-H); 4.65 (s, 2 H, $C\underline{H}_2COCH_3$); 2.40 (d, J=1.1, 3 H, CH_3); 2.39 (s, 3 H, $CH_2COC\underline{H}_3$).

6-Bromo-4,9-dimethyl-2H-furo[2,3-h]-1-benzopyran-2-one (23). A 4% ethanolic potassium hydroxide solution (30 mL) was slowly added to a solution of **22** (0.6 g, 2.0 mmol) in absolute ethanol (100 mL), and the mixture was refluxed in the dark for 1 h. The solution was cooled, diluted with water (100 mL) and acidified with diluted HCl. The ethanol was removed by evaporation under reduced pressure and the residue was extracted with $CHCl_3$ (3 x 75 mL). The organic phase was evaporated under reduced pressure and the solid residue was purified by column chromatography (eluent: $CHCl_3$) to give **23** (0.2 g, yield 34%); mp 220 °C. ^{1}H-NMR ($CDCl_3$): 7.64 (s, 1 H, 5-H); 7.50 (q, J=1.3, 1 H, 8-H); 6.28 (q, J=1.1, 1 H, 3-H); 2.53 (d, J=1.3, 3 H, 9-CH_3); 2.48 (d, J=1.1, 3 H, 4-CH_3).

***4,9-Dimethyl-6-phenyl-2H-furo[2,3-h]-1-benzopyran-2-one* (*24, 6-PhDMA*).** To a solution of **23** (0.3 g, 1.0 mmol) in DME (3 ml), phenylboronic acid (1.3 mmol) was added, followed by 1M Na_2CO_3 solution (3 ml) and palladium-tetrakis(triphenylphosphine) (5 mg, 4 µmol). The mixture was microwave irradiated at 130 °C (power set point 150W; ramp time 1 minute; hold time 5 minutes). After cooling, the mixture was stirred at RT until a precipitate was formed. The solid was collected by filtration, thoroughly washed with ethanol, and then dried under vacuum, affording **24** (0.2 g, yield 69%); mp 199 °C. ^{1}H-NMR (DMSO-d_6): 7.96 (q, J=1.3, 1 H, 8-H); 7.91-7.86 (m, 2 H, 2'-H, 6'-H); 7.77 (s, 1 H, 5-H); 7.59-7.51 (m, 2 H, 3'-H, 5'-H); 7.49-7.42 (m, 1 H, 4'-H); 6.41 (q, J=1.1, 1 H, 3-H); 2.58 (d, J=1.1, 3 H, 4-CH_3); 2,47 (d, J=1.3, 3 H, 9-CH_3). HRMS (ESI-TOF) for $C_{19}H_{15}O_3$ $[M + H]^+$: calcd.: 291.1021, found: 291.0450. Anal. calcd. for $C_{19}H_{14}O_3$: C, 78.61; H 4.86; found: C, 78.59; H, 4.86.

***4,9-Dimethyl-6-(4'-aminophenyl)-2H-furo[2,3-h]-1-benzopyran-2-one* (*25, pANDMA*).** To a solution of **23** (0.3 g, 1.0 mmol) in DME (3 ml), 4-aminophenylboronic acid pinacolester (0.3 g, 1.3 mmol) was added, followed by 1M Na_2CO_3 solution (3 ml) and palladium-tetrakis(triphenylphosphine) (5 mg, 4 µmol). The mixture was refluxed for 12 h, then cooled and stirred at RT until a precipitate was formed. The solid was collected by filtration and then purified by column chromatography (eluent: $CHCl_3$) to give **25** (0.1 g, yield 52%); mp 247 °C. ^{1}H-NMR (DMSO-d_6): 7.92 (q, J=1.3, 1 H, 8-H); 7.62 (s, 1 H, 5-H); 7.61-7.56 (m, 2 H, 2'-H, 6'-H); 6.73-6.68 (m, 2 H, 3'-H, 5'-H); 6.36 (q, J=1.1, 1 H, 3-H); 5.36 (s, 2 H, NH_2); 2.55 (d, J=1.3, 3 H, 9-CH_3); 2.45 (d, J=1.1,

3 H, 4-CH_3). HRMS (ESI-TOF) for $C_{19}H_{16}NO_3$ $[M + H]^+$: calcd.: 306.1130, found: 306.0754. Anal. calcd. for $C_{19}H_{15}NO_3$: C, 74.74; H 4.95; N, 4.59; found: C, 74.78; H 4.92; N, 4.55.

The **mesylate salt** was prepared as follows: to a solution of **25** in the minimum amount of THF methanesulphonic acid was added until complete precipitation. The solid was collected by filtration, washed with cold THF and dried under vacuum to give the mesylate salt. HRMS (ESI-TOF) for $C_{19}H_{16}NO_3$ $[M + H]^+$: calcd.: 306.1130, found: 306.0463. Anal. calcd. for $C_{20}H_{19}NO_6S$: C, 59.83; H 4.77; N, 3.49; found: C, 59.88; H 4.72; N, 3.48.

***4,9-Dimethyl-6-(3'-aminophenyl)-2H-furo[2,3-h]-1-benzopyran-2-one* (*26, mANDMA*).** To a solution of **23** (0.3 g, 1.0 mmol) in DME (3 ml), 3-aminophenylboronic acid (0.2 g, 1.3 mmol) was added, followed by 1M Na_2CO_3 solution (3 ml) and palladium-tetrakis(triphenylphosphine) (5 mg, 4 μmol). The mixture was refluxed for 2 h, then cooled and stirred at RT until a precipitate was formed. The solid was collected by filtration and then purified by column chromatography (eluent: $CHCl_3$) to give **26** (0.15 g, yield 49%); mp 265 °C. ^{1}H-NMR (DMSO-d_6): 7.94 (q, J=1.3, 1 H, 8-H); 7.66 (s, 1 H, 5-H); 7.16 (t, J=7.7, 1 H, 5'-H); 7.05 (t, J=2.0, 1 H, 2'-H); 6.96 (dt, J=7.5, J=1.3, 1 H, 6'-H); 6.66-6.60 (m, 1 H, 4'-H); 6.40 (q, J=1.1, 1 H, 3-H); 5.23 (s, 2 H, NH_2); 2.55 (d, J=1.3, 3 H, 9-CH_3); 2.46 (d, J=1.1, 3 H, 4-CH_3). HRMS (ESI-TOF) for $C_{19}H_{16}NO_3$ $[M + H]^+$: calcd.: 306.1130, found: 306.0415. Anal. calcd. for $C_{19}H_{15}NO_3$: C, 74.74; H 4.95; N, 4.59; found: C, 74.73; H 4.98; N, 4.56.

***4,9-Dimethyl-6-(4'-pyridyl)-2H-furo[2,3-h]-1-benzopyran-2-one* (*27, PyDMA*).** To a solution of **23** (0.3 g, 1.0 mmol) in DME (3 ml), 4-pyridylboronic acid (0.16 g, 1.3 mmol) was added, followed by 1M Na_2CO_3 solution (3 ml) and palladium-tetrakis(triphenylphosphine) (5 mg, 4 μmol). The mixture was microwave irradiated at 150 °C (power set point 200W; ramp time 1 minute; hold time 5 minutes). After cooling, the mixture was poured in water (50 mL) and the precipitate was collected by filtration. The solid was dissolved in HCl 5M (pH 5), and the acid solution was extracted with EtOAc (3 x 10 mL). The aqueous phase was alkalinized to pH 8 with NaOH 1M and extracted with EtOAc (3 x 10 mL). The organic phase was finally evaporated under reduced pressure obtaining **27** (0.1 g, yield 44%); mp 247 °C. ^{1}H-NMR (DMSO-d_6): 8.74 (dd, J=4.5, J=1.8, 2H, 3'-H, 5'-H); 8.0 (q, J=1.4, 3H, 8-H); 7.96 (dd, J=4.5, J=1.8, 2H, 2'-H, 4'-H); 7.95 (s, 1H, 5-H); 6.44 (q, J=1.4, 3H, 3-H); 2.60 (d, J=1.4, 3H, 9-CH_3); 2.48 (d, J=1.4, 3H, 4-CH_3). HRMS (ESI-TOF) for $C_{18}H_{14}NO_3$ $[M + H]^+$: calcd.: 292.0974, found: 292.0562. Anal. calcd. for $C_{18}H_{13}NO_3$: C, 74.22; H 4.50; N, 4.81; found: C, 74.20; H 4.52; N, 4.85.

***4,9-Dimethyl-6-(2'-thienyl)-2H-furo[2,3-h]-1-benzopyran-2-one* (*28, ThiDMA*).** To a solution of **23** (0.3 g, 1.0 mmol) in DME (3 ml), 2-thienylboronic acid (0.17 g, 1.3 mmol) was added, followed by 1M Na_2CO_3 solution (3 ml) and palladium-tetrakis(triphenylphosphine) (5 mg, 4 μmol). The mixture was microwave irradiated at 150 °C (power set point 200W; ramp time 1 minute; hold time 5

minutes). After cooling, the mixture was poured in water (50 mL) and the precipitate was collected by filtration. The solid was crystallized from acetonitrile to give **28** (30 mg, yield 10%); mp 256 °C. ^{1}H-NMR (DMSO-*d$_6$*): 7.78 (dd, J=3.7, J=1.3, 1H, 3'-H); 7.68 (s, 1H, 5-H); 7.54 (q, J=1.5, 3H, 8-H); 7.39 (dd, J=5.0 J=1.3, 1H, 5'-H); 7.18 (dd, J=5.0, J=3.7, 1H, 4'-H); 6.3 (q, J=1.1, 3H, 3-H); 2.57 (d, J=1.5, 3H, 9-CH_3); 2.54 (d, J=1.1, 3H, 4-CH_3). HRMS (ESI-TOF) for $C_{17}H_{13}O_3S$ [M + H]$^+$: calcd.: 297.0585, found: 297.0912. Anal. calcd. for $C_{17}H_{12}O_3S$: C, 68.90; H 4.08; found: C, 68.92; H 4.05.

Synthesis of 4-alkyl-6-penyl-2*H*-furo[2,3-*h*]-1-benzopyran-2-ones 34-35 (C series).

4-Phenylresorcinol (29). To a solution of **20** (3.8 g, 20.0 mmol) in DME (40 ml), phenylboronic acid (3.2 g, 26.0 mmol) was added, followed by 1M Na_2CO_3 solution (60 ml) and palladium-tetrakis(triphenylphosphine) (92 mg, 80 µmoli). The mixture was microwave irradiated at 130 °C (power set point 150W; ramp time 1 minute; hold time 5 minutes). After cooling, the mixture was poured into water (300 mL) and acidified with HCl 2M to pH 5. The acid solution was extracted with EtOAc (3 x 100 mL) and the organic phase was evaporated under reduced pressure. The solid residue was purified by column chromatography (eluent: $CHCl_3$) to give **29** (1.0 g, yield 26%); mp 147 °C. ^{1}H-NMR (DMSO-*d$_6$*): 9.35 (s, 1 H, OH); 9.31 (s, 1 H, OH); 7.48-7.44 (m, 2 H, 2'-H, 6'-H); 7.35-7.30 (m, 2 H, 3'-H, 5'-H); 7.22-7.17 (m, 1 H, 4'-H); 7.03 (d, J=8.3, 1 H, 5-H); 6.40 (d, J=2.4, 1 H, 2-H); 6.40 (dd, J=8.3, J=2.4, 1 H, 6-H).

7-Hydroxy-4-isopropyl-6-phenylbenzopyran-2-one (30). To an ice-cold solution of **29** (0.4 g, 2.0 mmol) in ethyl isobutyrylacetate (0.3 mL, 2.0 mmol), H_2SO_4 (2 mL) was added dropwise and the mixture was stirred at room temperature for 1 h. The resulting solution was poured into an ice/water mixture (50 g) and the obtained precipitate was filtered and washed with water to give **30** (140 mg, yield 25%); mp 187°C. ^{1}H-NMR ($CDCl_3$): 7.57-7.48 (m, 6 H, 5-H, Ar-H); 6.98 (s, 1 H, 8-H); 6.21 (s, 1 H, 3-H); 3.30-3.19 (m, 1 H, C$\underline{H}$(CH_3)$_2$); 1.31 (d, J=6.8, 6 H, CH(C$\underline{H}_3$)$_2$).

4-Cyclopropyl-7-hydroxy-6-phenylbenzopyran-2-one (31). To an ice-cold solution of **29** (0.4 g, 2.0 mmol) in ethyl 3-cyclopropyl-3-oxopropionate (0.3 mL, 2.0 mmol), H_2SO_4 (2 mL) was added dropwise and the mixture was stirred at room temperature for 1 h. The resulting solution was poured into an ice/water mixture (50 g) and the obtained precipitate was filtered and washed with water to give **31** (139 mg, yield 25%); mp 204°C. ^{1}H-NMR ($CDCl_3$): 7.76 (s, 1 H, 5-H); 7.58-7.46 (m, 5 H, Ar-H); 6.97 (s, 1 H, 8-H); 5.92 (s, 1 H, 3-H); 2.14-2.06 (m, 1 H, cyclopropyl-CH); 1.16-1.08 (m, 2 H, cyclopropyl-CH_2); 0.88-0.81 (m, 2 H, cyclopropyl-CH_2).

4-Isopropyl-7-(2'-oxopropyloxy)-6-penhylbenzopyran-2-one (32). A mixture of **30** (112 mg, 0.4 mmol), chloroacetone (48 µl, 0.6 mmol), KI (5 mg), and anhydrous K_2CO_3 (0.8 g, 6.0 mmol) in acetone (5 mL) was refluxed for 6 h. After cooling, the solid was filtered off and washed with fresh

acetone (3 mL). The solvent was evaporated from the combined filtrate and washings to give **32** (128 mg, yield 95%); mp 82 °C. ^{1}H-NMR ($CDCl_3$): 7.61 (s, 1 H, 5-H) 7.59-7.36 (m, 5 H, Ar-H); 6.73 (s, 1 H, 8-H); 6.23 (s, 1 H, 3-H); 4.60 (s, 2 H, $C\underline{H}_2COCH_3$); 2.20 (s, 3 H, $CH_2COC\underline{H}_3$); 3.32-3.24 (m, 1 H, $C\underline{H}(CH_3)_2$); 1.32 (d, J=6.6, 6 H, $CH(C\underline{H}_3)_2$ $CH(C\underline{H}_3)_2$).

4-Cyclopropyl-7-(2'-oxopropyloxy)-6-phenylbenzopyran-2-one (33). A mixture of **31** (111 mg, 0.4 mmol), chloroacetone (48 µl, 0.6 mmol), KI (5 mg), and anhydrous K_2CO_3 (0.8 g, 6.0 mmol) in acetone (5 mL) was refluxed for 6 h. After cooling, the solid was filtered off and washed with fresh acetone (3 mL). The solvent was evaporated from the combined filtrate and washings to give **33** (127 mg, yield 95%); mp 94 °C. ^{1}H-NMR ($CDCl_3$): 7.84 (s, 1 H, 5-H); 7.61-7.36 (m, 5 H, Ar-H); 6.72 (s, 1 H, 8-H); 5.94 (s, 1 H, 3-H); 4.61 (s, 2 H, $C\underline{H}_2COCH_3$); 2.19 (s, 3 H, $CH_2COC\underline{H}_3$); 2.11-2.03 (m, 1 H, cyclopropyl-CH); 1.17-1.10 (m, 2 H, cyclopropyl-CH_2); 0.89-0.82 (m, 2 H, cyclopropyl-CH_2).

4-Isopropyl-9-methyl-6-phenyl-2H-furo[2,3-h]-1-benzopyran-2-one (34, IPPhMA). A 4% ethanolic potassium hydroxide solution (5 mL) was slowly added to a solution of **32** (100 mg, 0.3 mmol) in absolute ethanol (20 mL), and the mixture was refluxed in the dark for 1 h. The solution was cooled, diluted with water (20 mL) and acidified with diluted HCl. The ethanol was removed by evaporation under reduced pressure and the residue was extracted with $CHCl_3$ (3 x 20 mL). The organic phase was evaporated under reduced pressure and the solid residue was purified by column chromatography (eluent: $CHCl_3$) to give **34** (19 mg, yield 20%); mp 148 °C. ^{1}H-NMR ($CDCl_3$): 7.81-7.77 (m, 2 H, 2'-H, 6'-H); 7.66 (s, 1 H, 5-H); 7.56-7.41 (m, 4 H, 3'-H, 4'-H, 5'-H, 8-H); 6.34 (s, 1 H, 3-H); 3.4-3.39 (m, 1 H, $C\underline{H}(CH_3)_2$); 1.58 (s, 3 H, 9-CH_3); 1.38 (d, J=6.8, 6 H, $CH(C\underline{H}_3)_2$). HRMS (ESI-TOF) for $C_{21}H_{19}O_3$ $[M + H]^+$: calcd.: 319.1334, found: 319.0669. Anal. calcd. for $C_{21}H_{18}O_3$: C, 79.23; H 5.70; found: C, 20.70; H, 5.68.

4-Cyclopropyl-9-methyl-6-phenyl-2H-furo[2,3-h]-1-benzopyran-2-one (35, CPPhMA). A 4% ethanolic potassium hydroxide solution (5 mL) was slowly added to a solution of **33** (100 mg, 0.3 mmol) in absolute ethanol (20 mL), and the mixture was refluxed in the dark for 1 h. The solution was cooled, diluted with water (20 mL) and acidified with diluted HCl. The ethanol was removed by evaporation under reduced pressure and the residue was extracted with $CHCl_3$ (3 x 20 mL). The organic phase was evaporated under reduced pressure and the residue was purified by column chromatography (eluent: $CHCl_3$) to give **35** (21 mg, yield 22%); mp 187°C. ^{1}H-NMR ($CDCl_3$): 7.91 (s, 1 H, 5-H); 7.84-7.80 (m, 2 H, 2'-H, 6'-H); 7.56-7.40 (m, 4 H, 3'-H, 4'-H, 5'-H, 8-H) 6.07 (s, 1 H, 3-H); 2.57 (d, J=1.3, 3 H, 9-CH_3); 2.19-2.11 (m, 1 H, cyclopropyl-CH); 1.26-1.16 (m, 2 H, cyclopropyl-CH_2); 0.92-0.86 (m, 2 H, cyclopropyl-CH_2). HRMS (ESI-TOF) for $C_{21}H_{17}O_3$ $[M + H]^+$: calcd.: 317.1178, found: 317.0792. Anal. calcd. for $C_{21}H_{16}O_3$: C, 79.73; H 5.10; found: C, 79.75; H, 5.08.

MASS SPECTRA OF FINAL COMPOUNDS

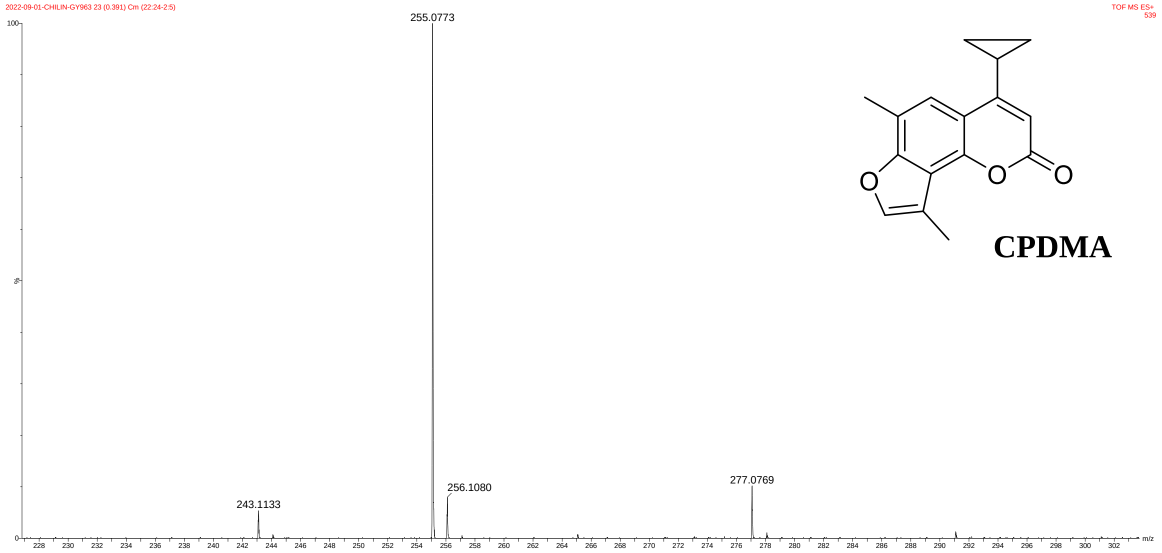

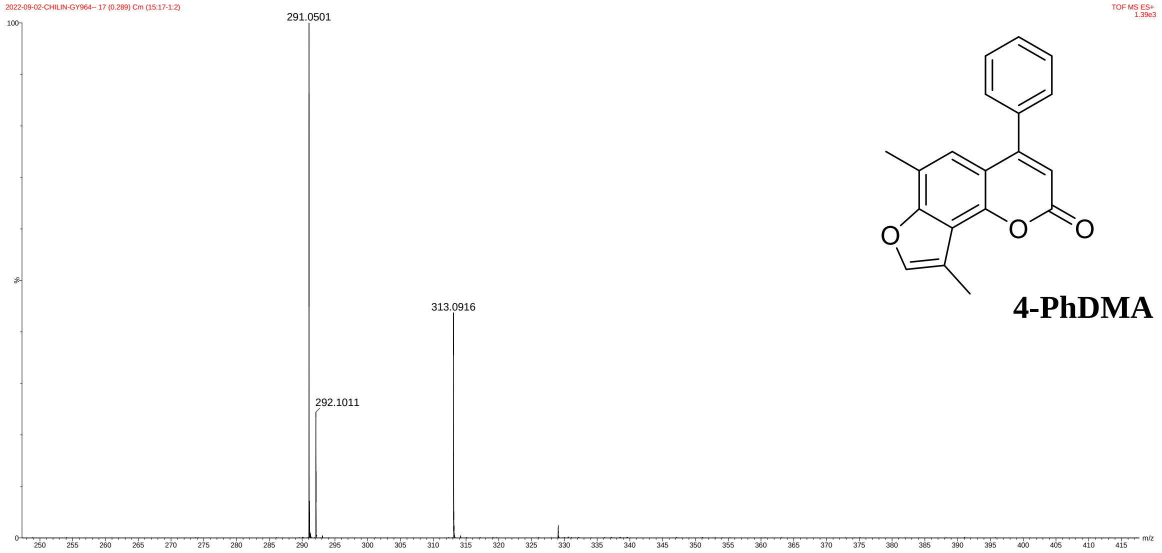

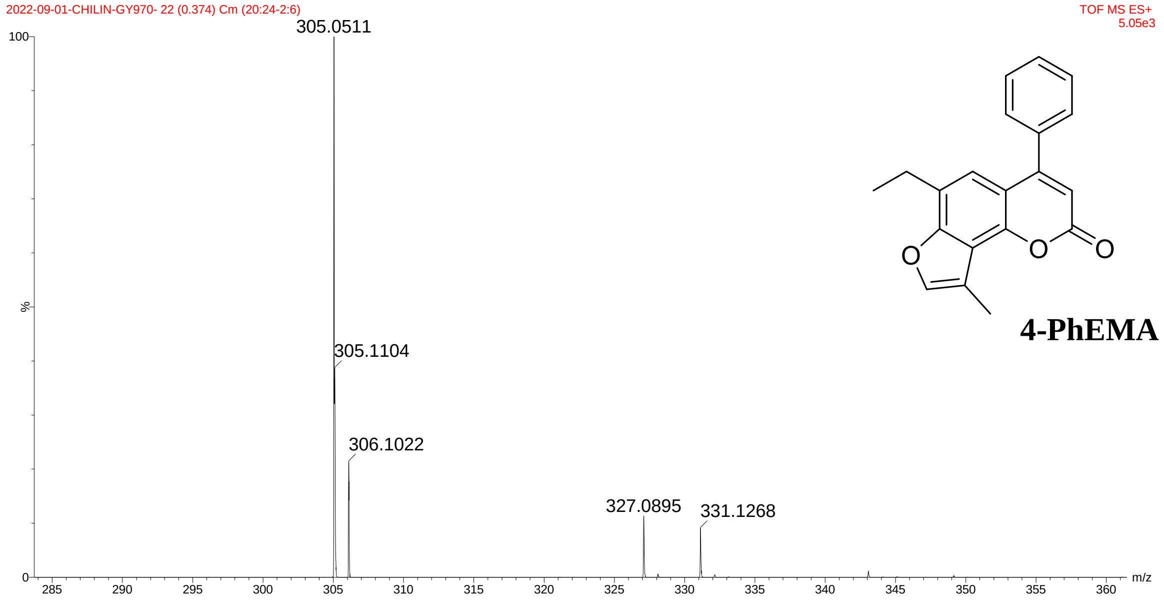

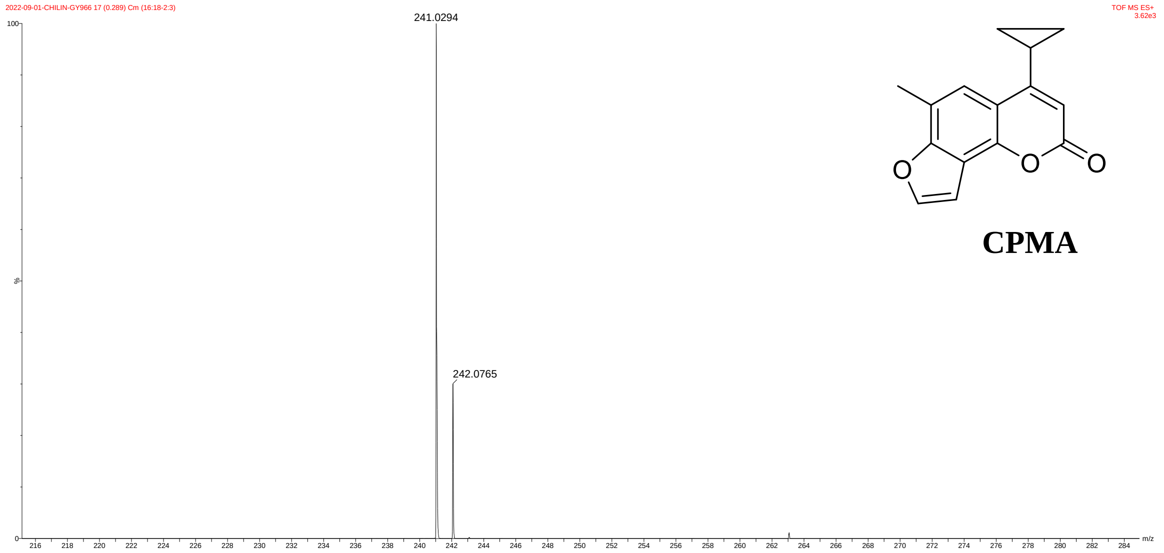

CPMA

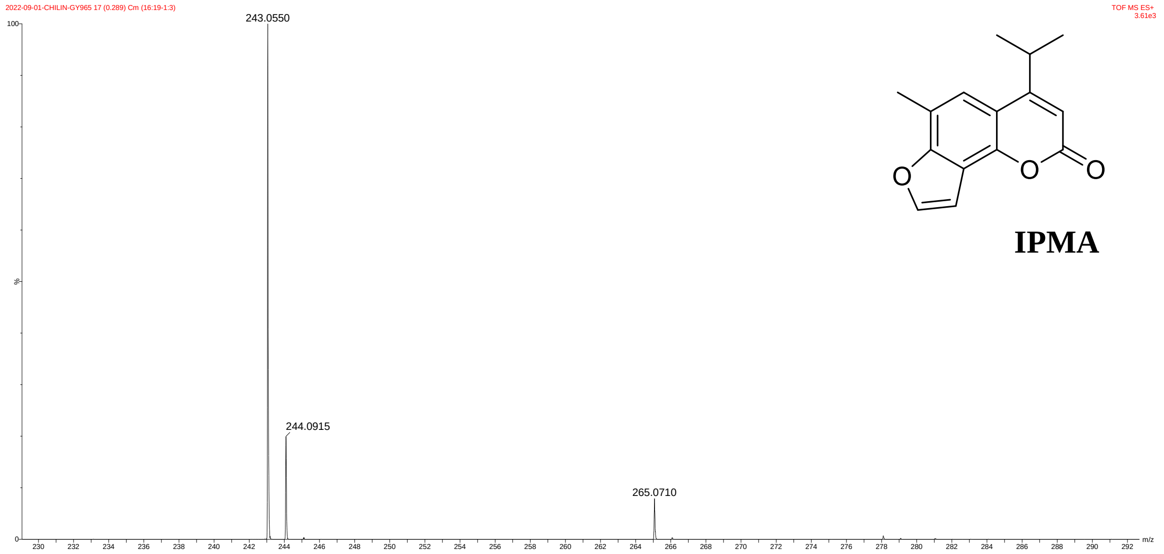

IPMA

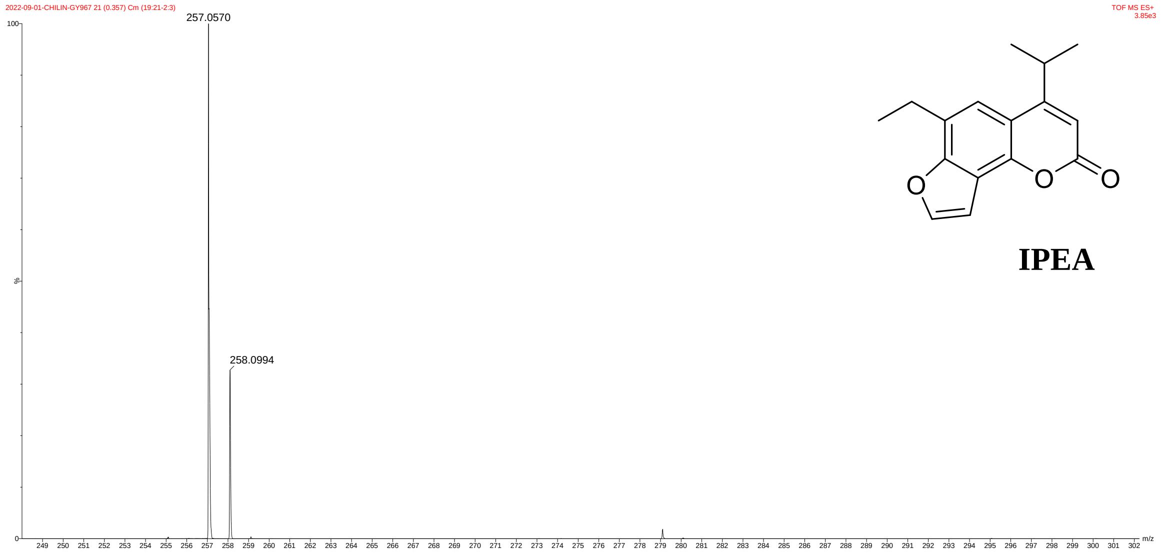

IPEA

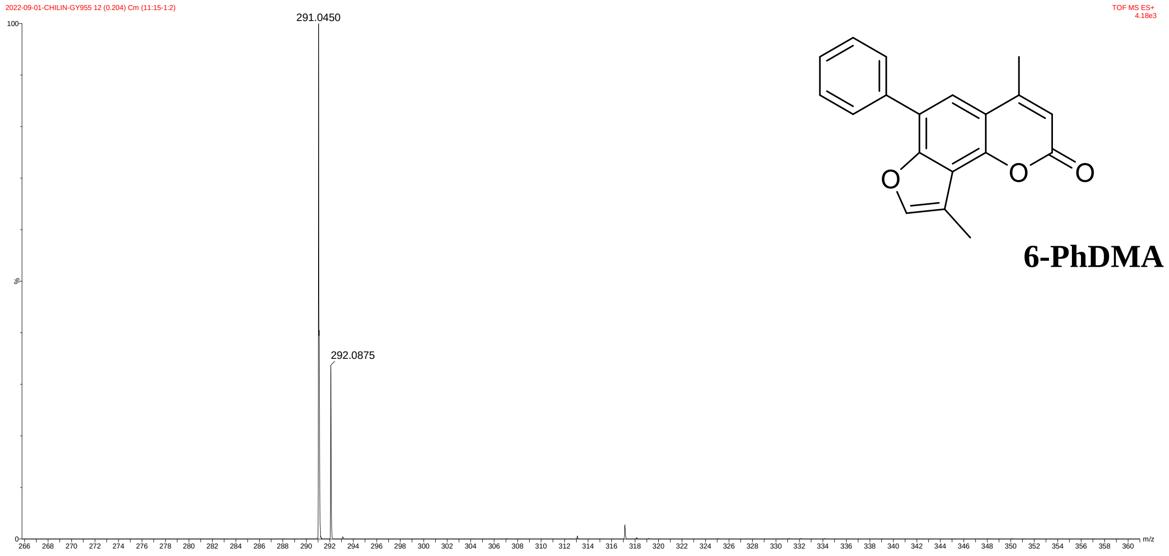
2022-09-01-CHILIN-GY955 12 (0.204) Cm (11:15-1:2)
TOF MS ES+
4.18e3
291.0450
292.0875
m/z
6-PhDMA

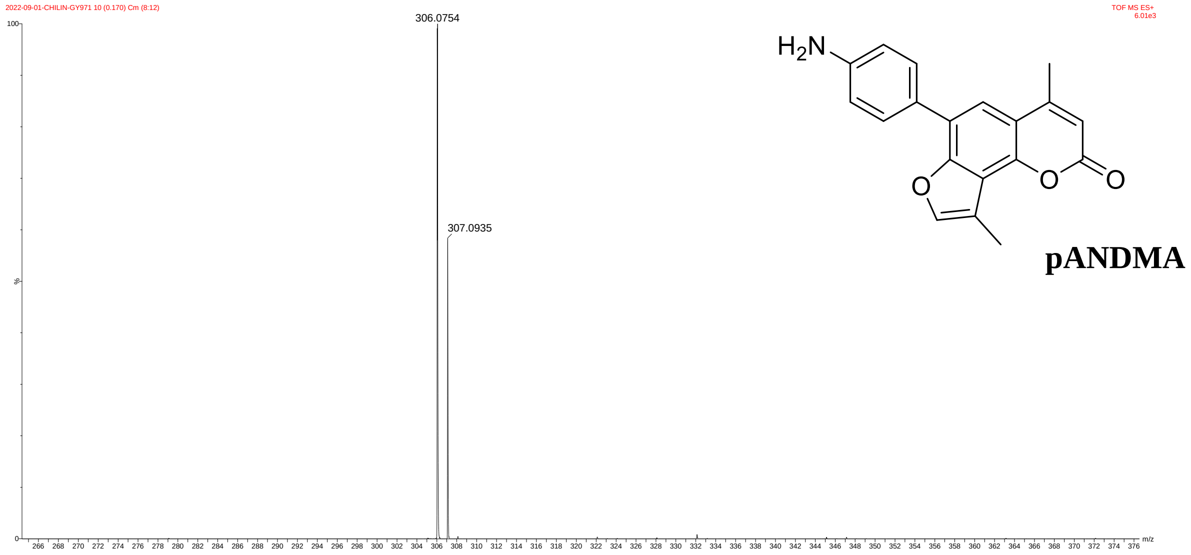
2022-09-01-CHILIN-GY971 10 (0.170) Cm (8:12)
TOF MS ES+
6.01e3
306.0754
307.0935
m/z
pANDMA

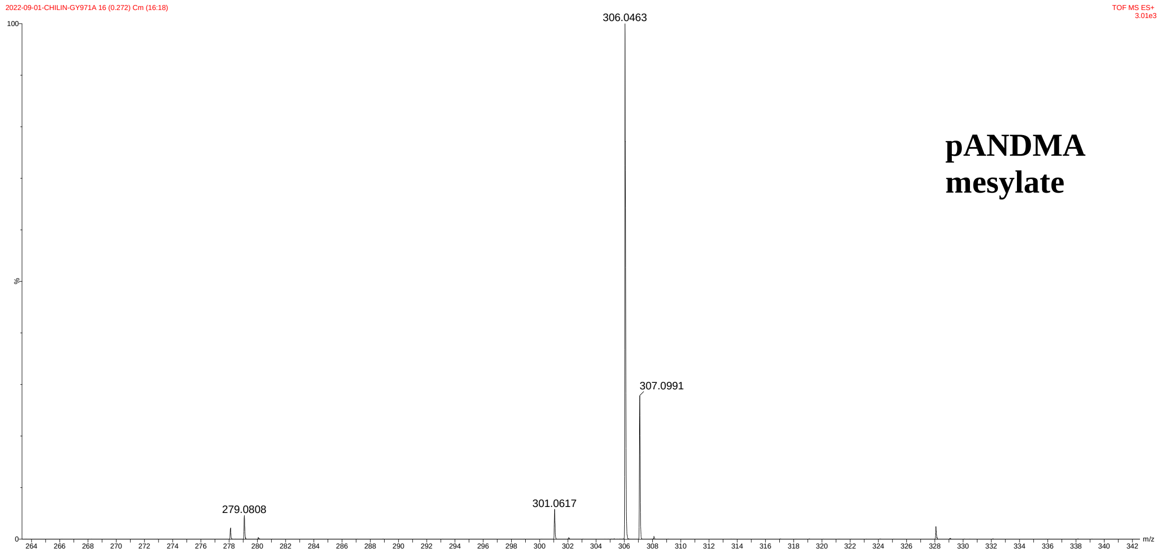
2022-09-01-CHILIN-GY971A 16 (0.272) Cm (16:18)
TOF MS ES+
3.01e3
306.0463
307.0991
279.0808
301.0617
m/z
pANDMA mesylate

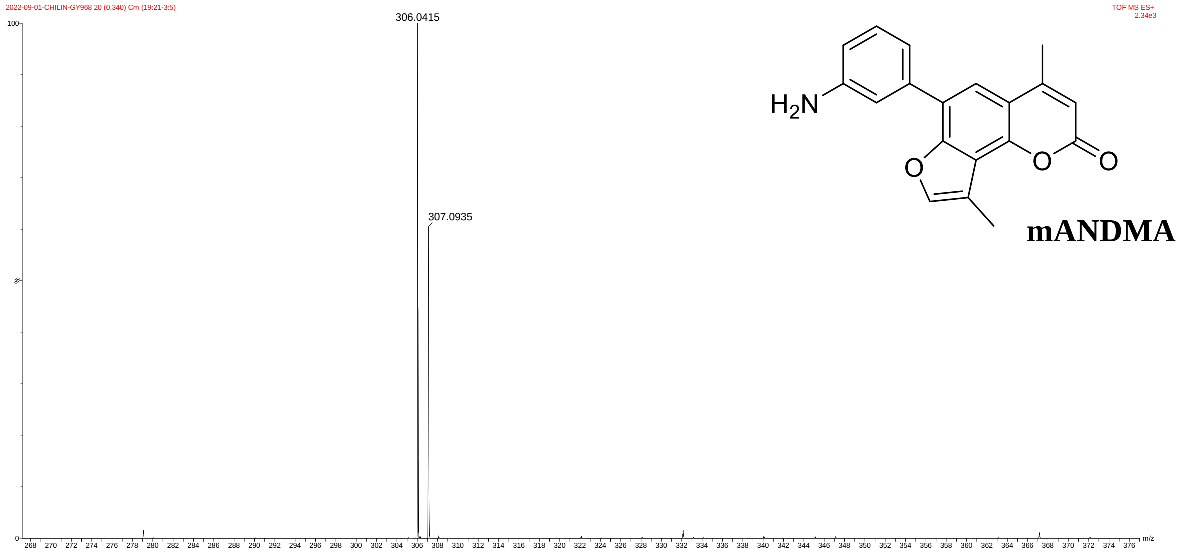
2022-09-01-CHILIN-GY968 20 (0.340) Cm (19:21-3:5)
TOF MS ES+
2.34e3
306.0415
307.0935
m/z
mANDMA

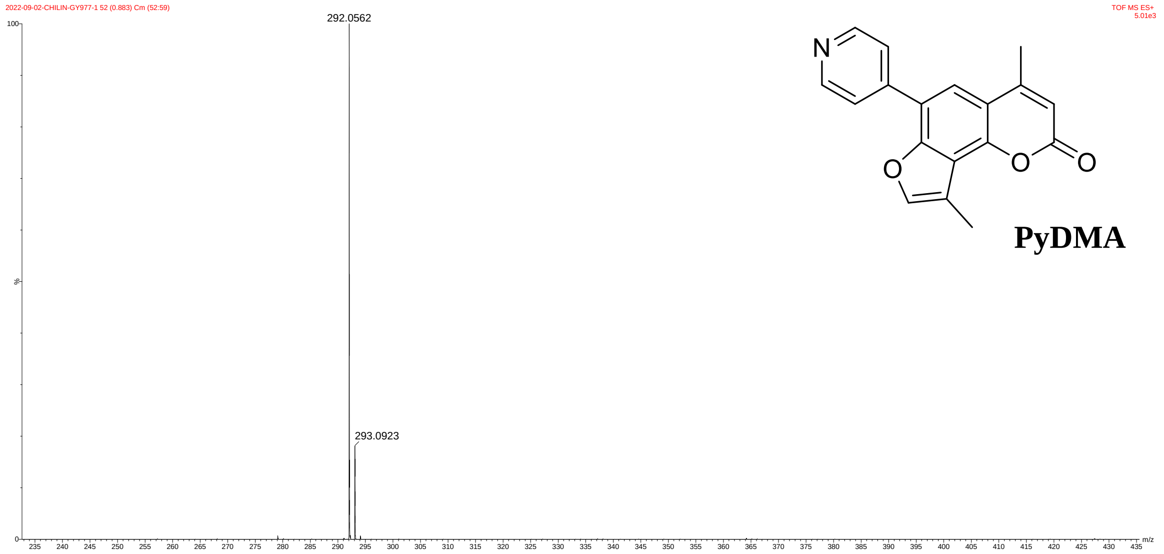
2022-09-02-CHILIN-GY977-1 52 (0.883) Cm (52:59)
TOF MS ES+
5.01e3
292.0562
293.0923
m/z
PyDMA

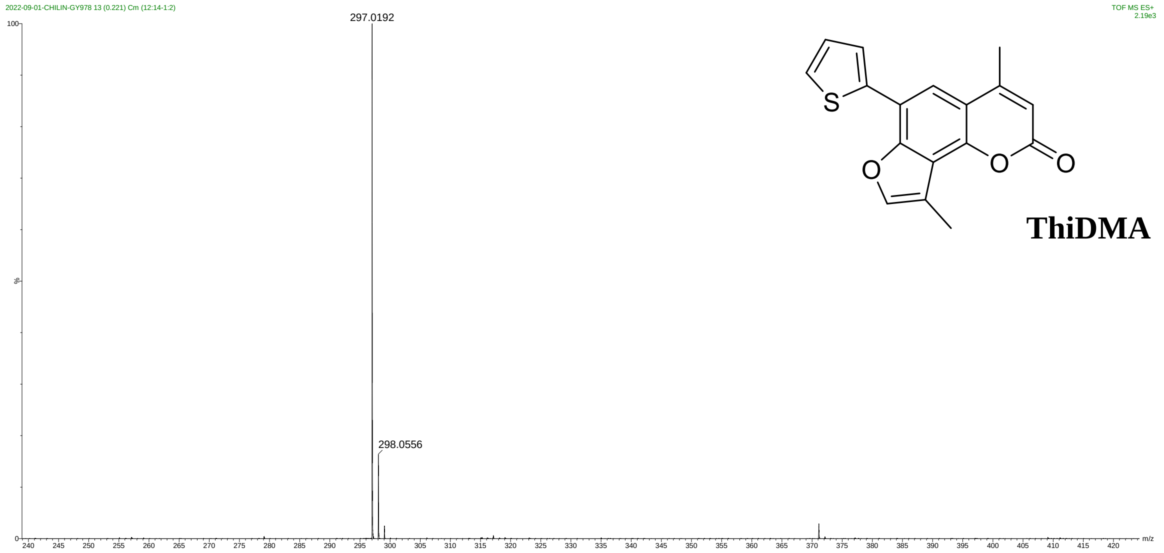
2022-09-01-CHILIN-GY978 13 (0.221) Cm (12:14-1:2)
TOF MS ES+
2.19e3
297.0192
298.0556
m/z
ThiDMA

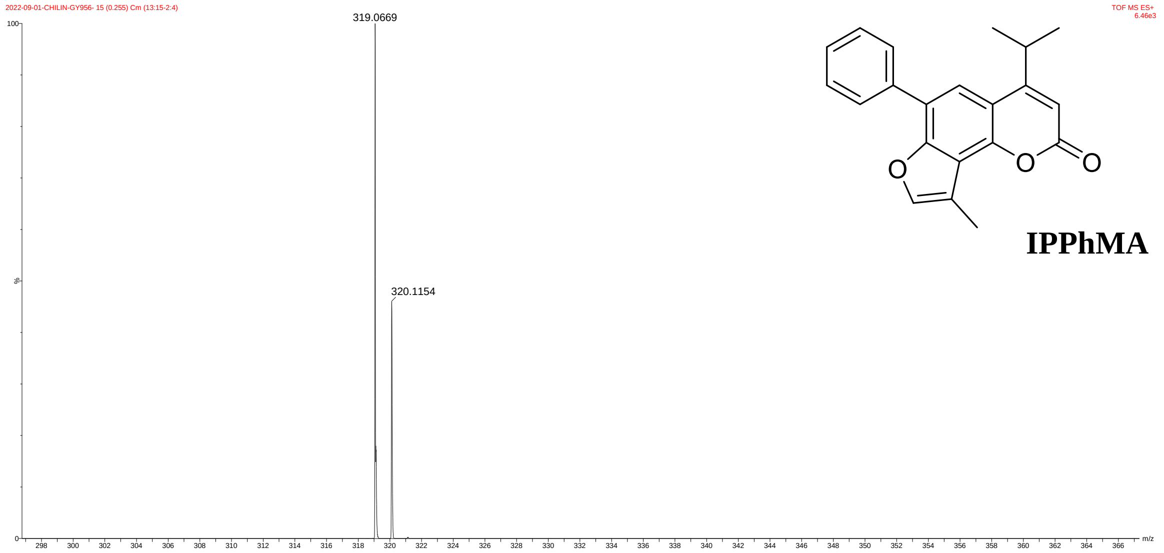

IPPhMA

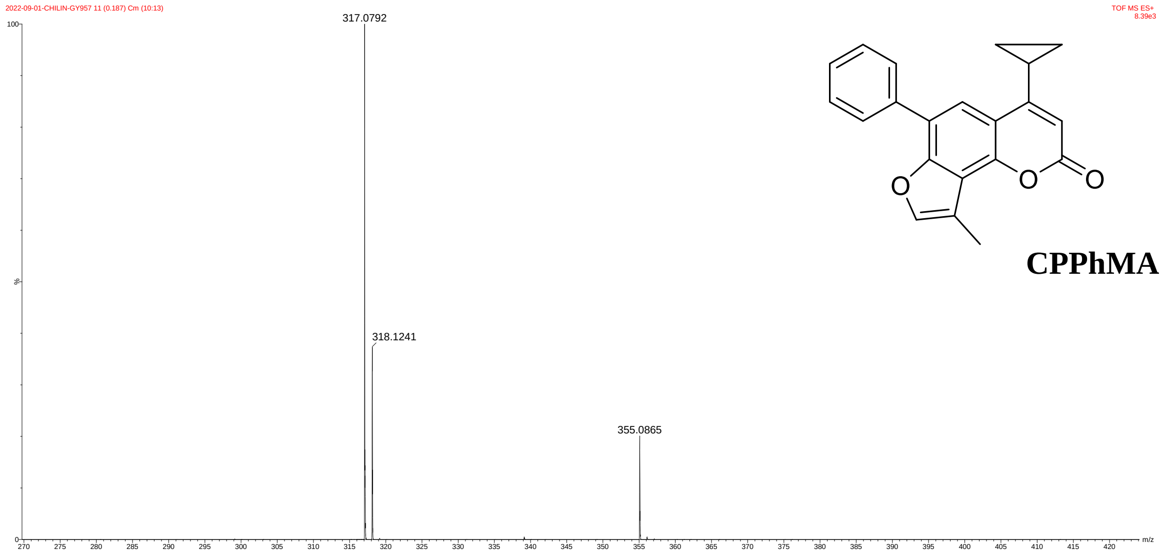

CPPhMA

BIOLOGY

EFFECT OF FINAL COMPOUNDS ON CFTR POTENTIATION

The effect of TMA analogues on CFTR potentiation was tested on CFBE41o- overexpressing wt CFTR and the high sensitivity halide-sensing yellow fluorescent protein (HS-YFP) YFP-H148Q/I152L (a generous gift from N. Pedemonte, Gaslini Institute, Genova, Italy). Cells were incubated with stimulation cocktail (20 μM forskolin and vehicle or 50 μM genistein, 1μM VX770, 200 nM TMA or TMA analogues) in the presence or absence of CFTRInh-172 for 30 min. The cover slips were then transferred onto a Nikon TMD inverted microscope, through a Nikon Fluor 40 objective, and signal was acquired with a Hamamatsu C2400–97 charge-coupled intensified video camera at a rate of 1 frame/3 s. Fluorescence coming from each single cell of at least 5 cells per field was analyzed by a customized software (Spin, Vicenza, Italy). Results are presented as transformed data to obtain the percentage signal variation ΔF(t) relative to the time of addition of stimulus, according to the equation: $\Delta F(t)=100[F(t)-F(0)]/F(0)$ where Ft and F0 are the fluorescence values at the time t, and at the time of iodide addition, respectively. YFP fluorescence decay rate was calculated by fitting fluorescence data of time courses by means of an exponential function. CFTR activity was calculated as the difference between YFP fluorescence decay rate in absence or in the presence of CFTRInh-172. None of the TMA analogues potentiate CFTR activity.

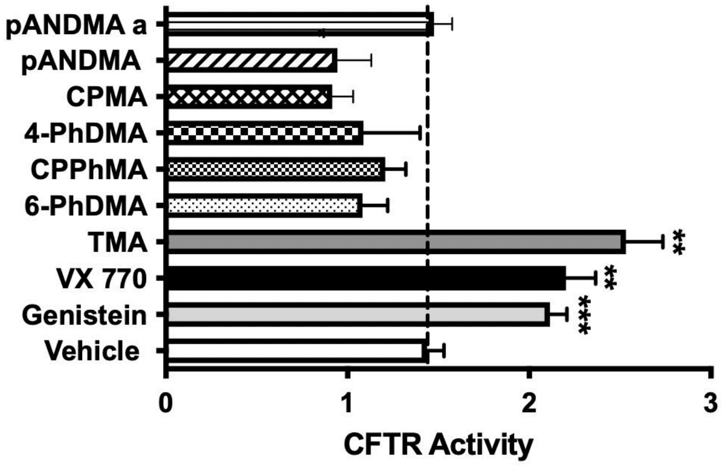

Figure S1. Evaluation of different TMA analogues as CFTR potentiators in CFBE41o- YFP- wt CFTR cells. Each bar corresponds to the mean ± SEM of data points coming from at least three different experiments (5–10 different cells/each experiment). Statistical comparisons were made using a nonparametric Kruskall-Wallis test (**$p < 0.01$, ***$p < 0.001$).

PHOTOTOXICITY

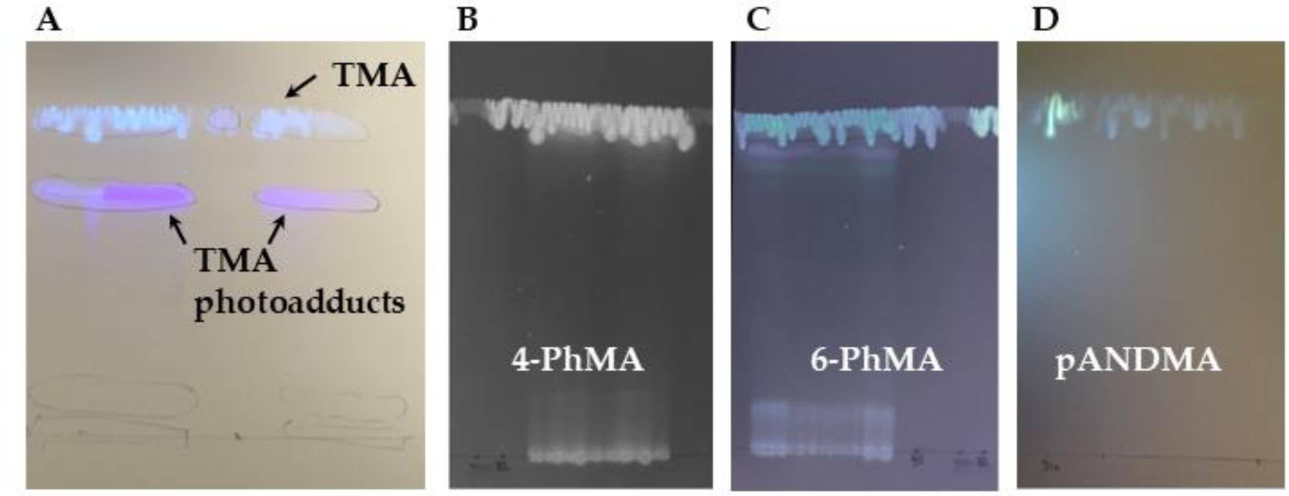

Figure S2. TLC and image analysis under UV light to evaluate the presence of photoadducts: TMA (A), 4-PhDMA (B), 6-PhDMA (C) and pANDMA (D).

MUTAGENICITY ASSAY ON *SALMONELLA TYPHIMRIUM* STRAINS.

Table S1. 4-PhDMA

	Salmonella typhimurium **TA97A**						*Salmonella typhimurium* **TA98**						*Salmonella typhimurium* **TA100**						*Salmonella typhimurium* **1535**					
	**- S9**			**+ S9**			**- S9**			**+ S9**			**- S9**			**+ S9**			**- S9**			**+ S9**		
	**average**	**SD**	**t/c**	**average**	**SD**	**t/c**	**average**	**SD**	**t/c**	**average**	**SD**	**t/c**	**average**	**SD**	**t/c**	**average**	**SD**	**t/c**	**average**	**SD**	**t/c**	**average**	**SD**	**t/c**
**DMSO**	77.3	7.2	1.0	109.0	20.2	1.0	39.0	4.6	1.0	46.0	4.4	1.0	195.7	16.0	1.0	171.0	7.8	1.0	11.0	1.0	1.0	10.7	0.6	1.0
**C+**	312.0	11.3	4.0	404.0	11.3	3.7	167.0	29.7	4.3	208.0	19.8	4.5	824.0	192.3	4.2	811.0	97.6	4.7	218.0	19.8	19.8	241.0	9.9	22.6
**5**	79.5	2.1	1.0	87.0	11.3	0.8	39.0	2.8	1.0	47.0	1.4	1.0	208.5	0.7	1.1	159.5	40.3	0.9	15.0	11.3	1.4	10.5	2.1	1.0
**10**	82.5	3.5	1.1	117.0	8.5	1.1	42.0	2.8	1.1	49.0	2.8	1.1	198.5	13.4	1.0	193.0	11.3	1.1	13.0	8.5	1.2	13.5	2.1	1.3
**20**	82.0	12.7	1.1	98.0	2.8	0.9	38.5	0.7	1.0	52.5	3.5	1.1	207.0	0.0	1.1	193.5	3.5	1.1	5.5	0.7	0.5	11.0	2.8	1.0
**50**	82.5	0.7	1.1	94.0	1.4	0.9	41.0	2.8	1.1	47.5	6.4	1.0	184.5	30.4	0.9	187.5	2.1	1.1	10.0	5.7	0.9	10.5	3.5	1.0
**100**	82.5	4.9	1.1	109.5	3.5	1.0	41.0	1.4	1.1	47.5	0.7	1.0	179.0	2.8	0.9	197.0	26.9	1.2	7.0	2.8	0.6	11.0	2.8	1.0

*Significant values according to Ames computation (Maron & Ames, 1983); DMSO, C+: negative and positive controls respectively; SD: Standard Deviation; t/c: number of revertant colonies in treated plates/number of revertant colonies in untreated plates.

Table S2. 6-PhDMA

	Salmonella typhimurium **TA97A**						*Salmonella typhimurium* **TA98**						*Salmonella typhimurium* **TA100**						*Salmonella typhimurium* **1535**					
	**- S9**			**+ S9**			**- S9**			**+ S9**			**- S9**			**+ S9**			**- S9**			**+ S9**		
	**average**	**SD**	**t/c**	**average**	**SD**	**t/c**	**average**	**SD**	**t/c**	**average**	**SD**	**t/c**	**average**	**SD**	**t/c**	**average**	**SD**	**t/c**	**average**	**SD**	**t/c**	**average**	**SD**	**t/c**
**DMSO**	77.3	7.2	1.0	109.0	20.2	1.0	39.0	4.6	1.0	46.0	4.4	1.0	195.7	16.0	1.0	171.0	7.8	1.0	11.0	1.0	1.0	10.7	0.6	1.0
**C+**	312.0	11.3	4.0	404.0	11.3	3.7	167.0	29.7	4.3	208.0	19.8	4.5	824.0	192.3	4.2	811.0	97.6	4.7	218.0	19.8	19.8	241.0	9.9	22.6
**5**	75.0	2.8	1.0	116.5	6.4	1.1	37.5	2.1	1.0	45.5	2.1	1.0	186.5	26.2	1.0	182.0	11.3	1.1	13.5	13.4	1.2	13.5	4.9	1.3
**10**	69.5	2.1	0.9	92.5	7.8	0.8	41.0	1.4	1.1	49.5	2.1	1.1	220.0	18.4	1.1	178.5	9.2	1.0	14.5	6.4	1.3	12.5	9.2	1.2
**20**	86.5	6.4	1.1	99.0	19.8	0.9	38.0	4.2	1.0	46.5	12.0	1.0	172.0	14.1	0.9	184.5	10.6	1.1	7.5	3.5	0.7	10.0	0.0	0.9
**50**	79.5	6.4	1.0	107.0	18.4	1.0	43.5	0.7	1.1	46.0	0.0	1.0	202.5	38.9	1.0	178.0	19.8	1.0	8.0	0.0	0.7	10.0	1.4	0.9
**100**	88.5	14.8	1.1	101.0	5.7	0.9	40.0	4.2	1.0	45.0	2.8	1.0	185.5	4.9	0.9	203.0	56.6	1.2	9.0	5.7	0.8	14.0	4.2	1.3

*Significant values according to Ames computation (Maron & Ames, 1983); DMSO, C+: negative and positive controls respectively; SD: Standard Deviation; t/c: number of revertant colonies in treated plates/number of revertant colonies in untreated plates.

Table S3. pANDMA

	Salmonella typhimurium **TA97A**						*Salmonella typhimurium* **TA98**						*Salmonella typhimurium* **TA100**						*Salmonella typhimurium* **1535**					
	**- S9**			**+ S9**			**- S9**			**+ S9**			**- S9**			**+ S9**			**- S9**			**+ S9**		
	**average**	**SD**	**t/c**	**average**	**SD**	**t/c**	**average**	**SD**	**t/c**	**average**	**SD**	**t/c**	**average**	**SD**	**t/c**	**average**	**SD**	**t/c**	**average**	**SD**	**t/c**	**average**	**SD**	**t/c**
**DMSO**	77.3	7.2	1.0	109.0	20.2	1.0	39.0	4.6	1.0	46.0	4.4	1.0	195.7	16.0	1.0	171.0	7.8	1.0	11.0	1.0	1.0	10.7	0.6	1.0
**C+**	312.0	11.3	4.0	404.0	11.3	3.7	167.0	29.7	4.3	208.0	19.8	4.5	824.0	192.3	4.2	811.0	97.6	4.7	218.0	19.8	19.8	241.0	9.9	22.6
**5**	82.5	3.5	1.1	88.5	7.8	0.8	40.5	0.7	1.0	50.0	2.8	1.1	192.0	2.8	1.0	175.0	2.8	1.0	17.5	7.8	1.6	14.5	0.7	1.4
**10**	80.5	3.5	1.0	100.5	2.1	0.9	40.5	2.1	1.0	48.0	1.4	1.0	189.0	15.6	1.0	181.5	23.3	1.1	13.0	8.5	1.2	17.0	4.2	1.6
**20**	82.0	8.5	1.1	92.5	4.9	0.8	43.5	0.7	1.1	49.5	4.9	1.1	204.5	23.3	1.0	224.0	29.7	1.3	5.5	2.1	0.5	12.0	2.8	1.1
**50**	86.5	6.4	1.1	104.5	2.1	1.0	41.0	5.7	1.1	51.0	5.7	1.1	189.0	0.0	1.0	232.0	41.0	1.4	9.5	6.4	0.9	13.0	8.5	1.2
**100**	86.0	4.2	1.1	101.0	4.2	0.9	39.0	1.4	1.0	45.5	3.5	1.0	165.5	14.8	0.8	235.5	40.3	1.4	10.0	2.8	0.9	14.0	5.7	1.3

*Significant values according to Ames computation (Maron & Ames, 1983); DMSO, C+: negative and positive controls respectively; SD: Standard Deviation; t/c: number of revertant colonies in treated plates/number of revertant colonies in untreated plates.

CYTOTOXICITY ASSAY ON HepG2 CELLS

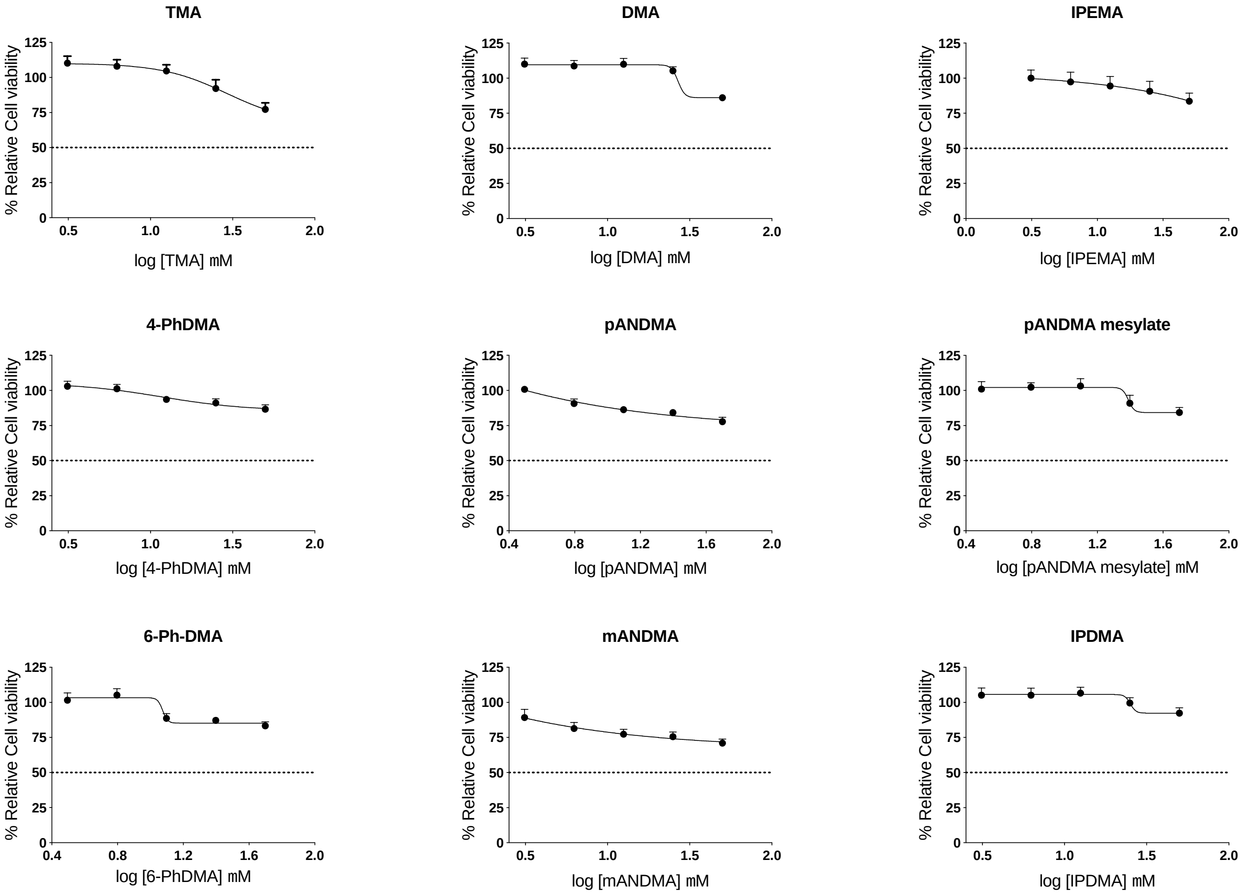

Figure S3. Cytotoxicity of a small library of select compounds on HepG2 cells. Only limited, non-significant drops of cell viability could be evidenced with all the tested molecules up to 50 μM. The data shown in this figure are the results of three independent experiments, each performed in quadruplicate.
